# Efficient cytosolic delivery of luminescent lanthanide bioprobes in live cells for two-photon microscopy†

**DOI:** 10.1039/d4sc00896k

**Published:** 2024-05-17

**Authors:** Kyangwi P. Malikidogo, Thibault Charnay, Daouda Ndiaye, Ji-Hyung Choi, Lucile Bridou, Baptiste Chartier, Sule Erbek, Guillaume Micouin, Akos Banyasz, Olivier Maury, Véronique Martel-Frachet, Alexei Grichine, Olivier Sénèque

**Affiliations:** a Univ. Grenoble Alpes, CNRS, CEA, IRIG, LCBM (UMR 5249) F-38000 Grenoble France olivier.seneque@cea.fr; b Univ. Grenoble Alpes, CNRS, DCM (UMR 5250) F-38000 Grenoble France; c Univ. Lyon, ENS de Lyon, CNRS UMR 5182, Laboratoire de Chimie Lyon F-69342 France; d Univ. Grenoble Alpes, INSERM U1209, CNRS UMR 5309, Institute for Advanced Biosciences F-38000 Grenoble France; e EPHE, PSL Research University 4-14 rue Ferrus 75014 Paris France

## Abstract

Lanthanide(iii) (Ln^3+^) complexes have desirable photophysical properties for optical bioimaging. However, despite their advantages over organic dyes, their use for microscopy imaging is limited by the high-energy UV excitation they require and their poor ability to cross the cell membrane and reach the cytosol. Here we describe a novel family of lanthanide-based luminescent probes, termed dTAT[Ln·L], based on (i) a DOTA-like chelator with a picolinate moiety, (ii) a two-photon absorbing antenna to shift the excitation to the near infrared and (ii) a dimeric TAT cell-penetrating peptide for cytosolic delivery. Several Tb^3+^ and Eu^3+^ probes were prepared and characterized. Two-photon microscopy of live cells was attempted using a commercial microscope with the three probes showing the highest quantum yields (>0.15). A diffuse Ln^3+^ emission was detected in most cells, which is characteristic of cytosolic delivery of the Ln^3+^ complex. The cytotoxicity of these three probes was evaluated and the IC_50_ ranged from 7 μM to >50 μM. The addition of a single positive or negative charge to the antenna of the most cytotoxic compound was sufficient to lower significantly or suppress its toxicity under the conditions used for two-photon microscopy. Therefore, the design reported here provides excellent lanthanide-based probes for two-photon microscopy of living cells.

## Abstract 2

We report a family of luminescent lanthanide bioprobes featuring a two-photon absorbing antenna and a dimer of the TAT cell penetrating peptides. They penetrate live cells efficiently and allow two-photon microscopy with commercial microscopes.

## Graphical abstract

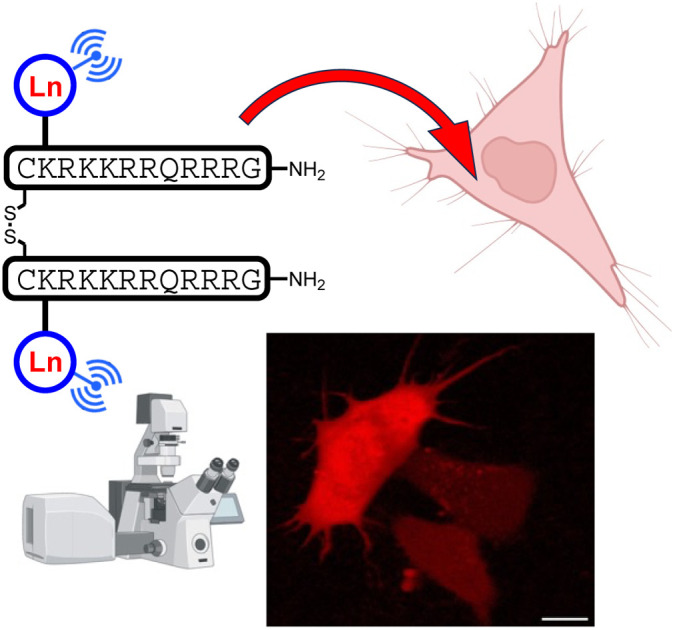

## Introduction

Fluorescence microscopy is an essential tool in biological research^1^ and among the various techniques, Laser Scanning Confocal Microscopy (LSCM), which allows imaging of a cell section and 3D reconstruction of cells, is one of the most popular techniques today.^2,3^ LSCM has prompted the development of genetically encoded fluorescent proteins or synthetic cell-permeable molecular fluorescent dyes to reveal subcellular components or monitor biological processes.^4–6^ The first fluorophores used for LSCM required high-energy excitation in the UV-blue optical window, which damages cells. This led to the development of two-photon microscopy (2PM),^7^ a technique based on two-photon (2P) excited fluorescence, in which excitation of the molecule is caused by simultaneous absorption of two photons of equal energy, half the energy required for single photon (1P) excitation. Consequently, instead of being excited in the UV-blue window, 2P-absorbing dyes are excited with near-infrared (NIR) light, which is less energetic and therefore less damaging to cells. In addition, NIR light penetrates deeper into the biological tissues.^8^ Since 2P excitation happens only in a very small volume corresponding to the focal point of the pulsed laser source, 2PM is comparable to LSCM in its ability to image cell sections, but superior to LSCM for imaging live cells or thick biological samples due to the NIR excitation. Over the past two decades, numerous organic fluorescent dyes have been developed that are suitable for 1P or 2P excitation in the red-NIR window, but in many cases they suffer from a lack of photostability, poor water solubility and low cell penetration capacity.^9–11^

Complexes of trivalent lanthanides (Ln^3+^) exhibit interesting luminescence properties for biological applications, making them an attractive alternative to organic fluorescent dyes.^12–17^ Each Ln^3+^ exhibits a fingerprint-like emission consisting of sharp atom-like emission bands at fixed wavelengths, independent of the environment, making them easily recognizable. Depending on the Ln^3+^, these bands cover the visible and NIR. Ln^3+^ luminescence is characterized by long lifetimes (in the microsecond to millisecond range), allowing background fluorescence (in the nanosecond range) to be suppressed by time-gated detection. In addition, luminescent Ln^3+^ complexes are highly photostable compared to organic dyes. They have been widely used in biological assays based on homogenous time-resolved technology.

Despite these advantages, Ln^3+^ complexes are not routinely used by biologists for cell microscopy as compared to organic dyes. There are two reasons for this. First, due to the low extinction coefficient of the f–f transitions, Ln^3+^ luminescence must rely on the antenna effect to be effective: a light-harvesting chromophore located close to the Ln^3+^ cation is excited and transfers energy to the Ln^3+^ to bring it into its emitting excited state. However, the antenna chromophore usually requires UV excitation which is damaging to cells. To overcome this problem, it is possible to use Ln^3+^ complexes with 2P-absorbing push–pull antennas.^18–20^ Importantly, it was demonstrated that 2PM with such Ln^3+^ complexes is possible with commercial microscopes commonly used by biologists.^21^ However, most of these 2P-absorbing Ln^3+^ complexes fail to penetrate live cells and cell fixation was required prior to incubation with the Ln^3+^ probe in order to permeabilize the cell membrane and let the probe go into the cell.

The second reason against using Ln^3+^ complexes as dyes for LCSM or 2PM is their cell penetration and their cellular localization properties. Parker and co-workers have studied the cell uptake and localization of various Ln^3+^ complexes with cyclen-based macrocyclic chelator with appended antenna. Many of them are able to penetrate cells, either neutral, negatively or positively charged. Indeed, cell penetration seems not to depend on charge but rather on antenna nature and linkage.^22–26^ Nevertheless, this cell uptake property does not apply to all Ln^3+^ complexes since other negatively charged cyclen-based Ln^3+^ complexes that we have recently described are unable to penetrate cell.^27^ Parker and co-workers have performed LSCM of cells using numerous Ln^3+^ complexes with 1P excitation in the UV.^26,28,29^ Most often, however, Ln^3+^ complexes end up in lysosomes or stick to the mitochondrial membrane.^26^ They are not properly delivered to the cytosol. Furthermore, it is difficult to predict the subcellular localization of a Ln^3+^ complex on the basis of its molecular structure, as localization can be influenced by minor changes in the antenna or chelator moieties. A convenient solution to these problems would be to conjugate the Ln^3+^ complex to a cell-penetrating peptide (CPP). CPPs are short peptides, usually cationic, that enter cells either by direct membrane translocation or by endocytosis.^30^ However, the CPP has to be chosen carefully, as many CPPs, whether natural such as TAT (transactivator of transcription of human immunodeficiency virus) or synthetic such as R8 (octa-arginine), do not effectively reach the cytosol.^31^

**Fig. 1:**
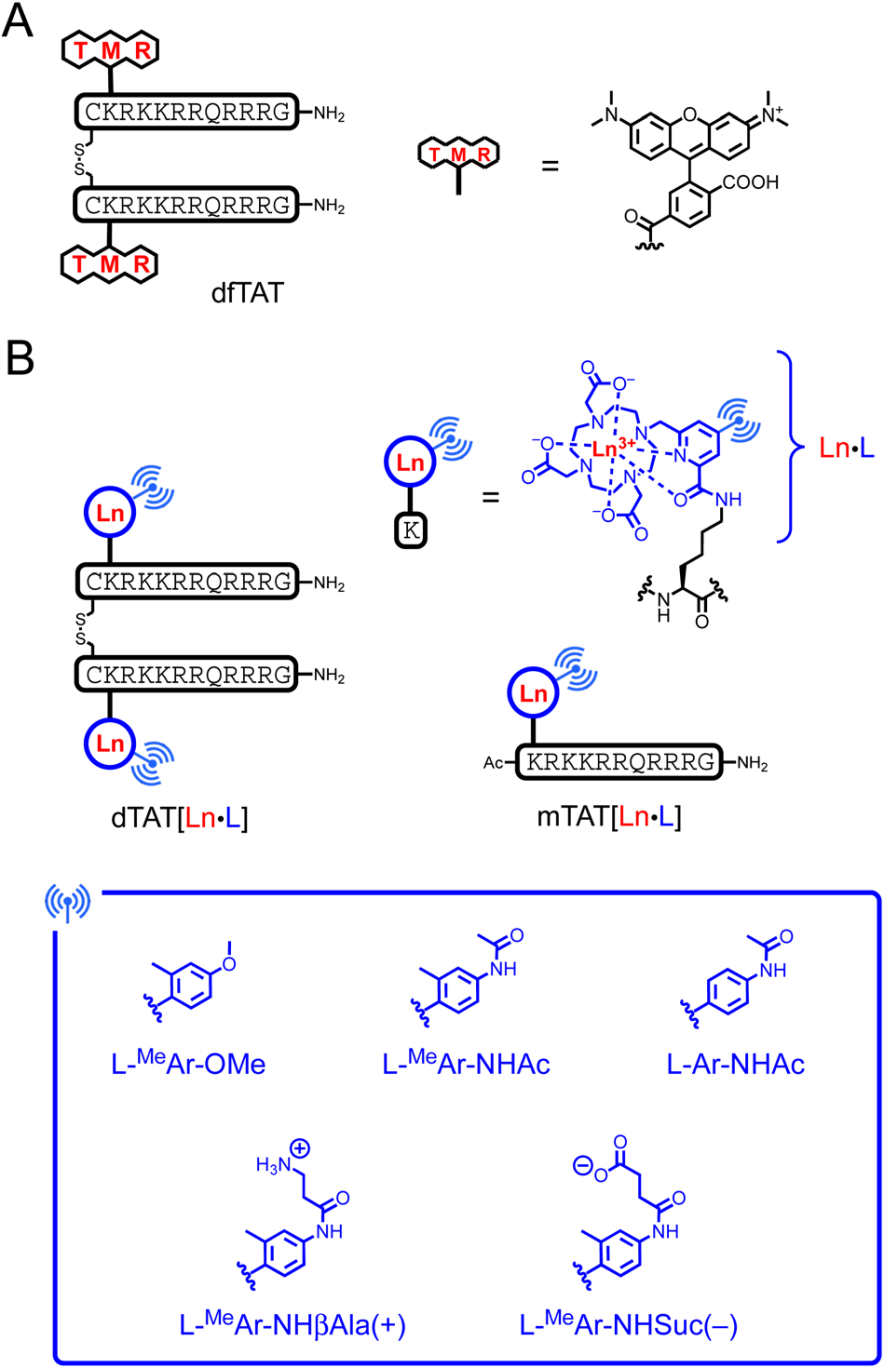
Chemical structure of (A) dfTAT and (B) Ln^3+^-based probes dTAT[Eu·L] and mTAT[Eu·L]. L denotes the DO3Apic macrocycle.

Ten years ago, Pellois and co-workers described a dimer of the TAT CPP, called dfTAT, with a (tetramethyl)rhodamine dye grafted onto each TAT monomer and dimerized by a disulfide bond (Fig. 1A).^32,33^ dfTAT is internalized into living cells by endocytosis and promotes endosomal leakage so that the rhodamine fluorophore reaches the cytosol and spreads throughout the entire cell. Inspired by this work, we describe here a family of luminescent bioprobes, called dTAT[Ln·L], consisting of Ln^3+^ complex with 2P absorbing push–pull antennas conjugated to a TAT dimer (Fig. 1B). We demonstrate that they enable 2PM imaging of living cells thanks to the appropriate photophysical properties of the Ln^3+^ complexes and the efficient cytosolic delivery with the TAT dimer. In addition, we show that toxicity issues can be easily controlled by modulation of the antenna.

## Results and discussion

### Design of the probes

Two factors are important for efficient cytosolic delivery of the dfTAT system (Fig. 1A): (i) the high number and high density of arginines in the dimer and (ii) the hydrophobic aromatic core of the rhodamine dye. Therefore, we decided to evaluate whether replacing the rhodamine dye with a Ln^3+^ complex could provide a probe that reaches the cytosol, with the aromatic sensitizing antenna playing the role of the rhodamine aromatic core in endosomal escape. As a Ln^3+^ ligand, we chose DO3Apic, a DOTA-type ligand in which one of the acetate arms has been replaced by a picolinate one. It forms stable complexes with Ln^3+^ that exhibit interesting luminescence properties, including long luminescence lifetimes and moderate to high quantum yields for Eu^3+^ and Tb^3+^.^34^ The addition of a π-conjugated system with an electron donating group to the picolinate antenna allows a red-shift of the excitation wavelength and an increase in the extinction coefficient for superior luminescence properties^27,35^ and converts the picolinate into a push–pull antenna suitable for 2P absorption.^35^ From a synthetic point of view, it is easy to distinguish the carboxylate of the picolinate from those of the acetates by playing with *tert*-butyl/methyl ester protection (see ESI†) in order to use it as a unique conjugation site for coupling to a peptide lysine side chain. Therefore, we decided to use the DO3Apic macrocycle as a Ln^3+^ ligand in the TAT dimer system. From our experience, we know that aryl-alkynyl-picolinate antennas, which are excellent for sensitizing Eu^3+^ luminescence, are not very stable under the conditions required for peptide synthesis (especially under the acidic conditions used for resin cleavage and deprotection steps) due to the addition of nucleophiles on the triple bond.^27^ Therefore, we opted for aryl-picolinate antennas and three of them were evaluated (Fig. 1B). The first one has an *ortho*-methyl group and a *para*-methoxy, which acts as an electrodonating group in the push–pull system. It was previously shown that such an antenna sensitizes Tb^3+^ better than Eu^3+^.^36^ In the second antenna, the methoxy group is replaced by an acetamide moiety and in the third antenna, the *ortho*-methyl group is deleted compared to the second one. In order to characterize the luminescence properties of the corresponding Tb^3+^ and Eu^3+^ complexes, the six conjugates mTAT[Ln·L-^Me^Ar-OMe], mTAT[Ln·L-^Me^Ar-NHAc] and mTAT[Ln·L-Ar-NHAc] with Ln = Tb and Eu (Fig. 1B) were prepared. The Gd^3+^ analogues were also prepared to determine the energy of their excited triplet state. Their synthesis is described in the ESI.†

### Photophysical properties

**Fig. 2:**
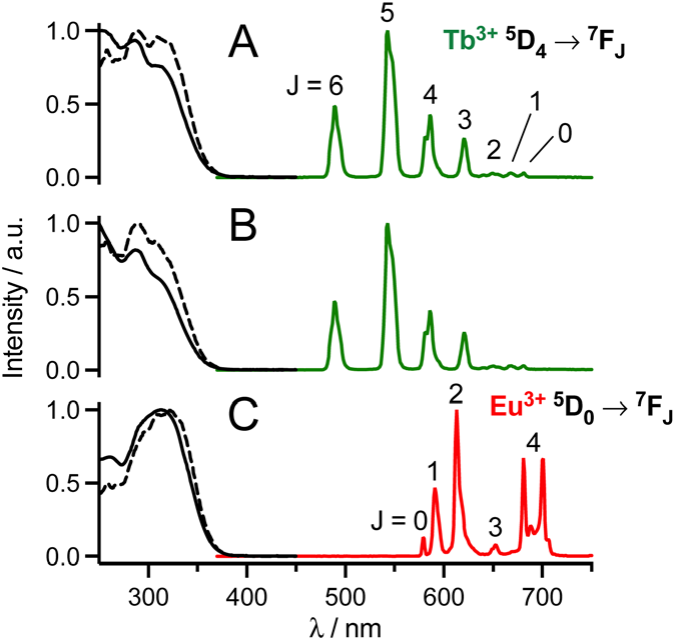
Normalized 1P absorption (black solid line), 1P excitation (black dashed line; *λ*_em_ = 545 nm (A and B) or 615 nm (C)) and emission (coloured solid line; *λ*_ex_ = 315 nm) spectra of (A) mTAT[Tb·L-^Me^Ar-OMe], (B) mTAT[Tb·L-^Me^Ar-NHAc] and (C) mTAT[Eu·L-Ar-NHAc] in PBS.

The luminescence properties of the probes were evaluated in PBS (pH 7.4). Absorption, excitation and emission spectra are shown in Fig. 2 or in the ESI† and spectroscopic data are summarized in Table 1. Absorption spectra of mTAT[Ln·L] compounds show two bands at *ca.* 285 and 315 nm, which are attributed to locally excited (LE) and intra-ligand charge transfer (ILCT) transitions within the antenna, respectively.^27,36^ In the case of twisted antennas, *i.e.* with an *ortho*-methyl group, the LE band dominates and the ILCT band appears as a shoulder while in the case of the freely rotating antenna, *i.e.* in the absence of *ortho*-methyl, the ILCT band dominates the absorption. The ILCT band extends up to *ca.* 365 nm (*λ*_cut-off_ in Table 1).

**Table 1:** Spectroscopic characterizations in aerated PBS and cytotoxicity (IC_50_) determined by MTT proliferation assay on HeLa cells^a^

Compound	*λ*_max_; *λ*_cut-off_/nm	*E*(S_1_); *E*(T_1_)^b^/cm^−1^	*ε* at *λ*_max_/M^−1^ cm^−1^	*σ*_2P_ at 700 nm/GM	*Φ*_Ln_	*τ*_Ln_/ms	IC_50_/μM
mTAT[Ln·L-^Me^Ar-OMe]	285; 363	S_1_: 27 600	16 000	5.7	Tb: 0.55	Tb: 2.11	
		T_1_: 22 900			Eu: 0.040	Eu: 1.05	
mTAT[Ln·L-^Me^Ar-NHAc]	287; 361	S_1_: 27 800	14 000	4.2	Tb: 0.20	Tb: 0.83	
		T_1_: 22 700			Eu: 0.048	Eu: 1.05	
mTAT[Ln·L-Ar-NHAc]	315; 366	S_1_: 27 300	20 000	11.1	Tb: 0.027	Tb: 0.07	
		T_1_: 22 000			Eu: 0.15	Eu: 1.05	
dTAT[Tb·L-^Me^Ar-OMe]	285, 366		32 000^a^		Tb: 0.38	Tb: 1.40	18 ± 3
dTAT[Tb·L-^Me^Ar-NHAc]	288, 364		28 000^c^		Tb: 0.12	Tb: 0.46 (98%), 1.63 (2%)	7.6 ± 1.6
dTAT[Eu·L-Ar-NHAc]	317; 370		40 000^c^		Eu: 0.15	Eu: 1.08	>50
dTAT[Tb·L-^Me^Ar-NHβAla(+)]	289, 366		30 000		Tb: 0.14	Tb: 0.34 (99%), 1,43 (1%)	22 ± 5
dTAT[Tb·L-^Me^Ar-NHSuc(−)]	286, 368		26 000		Tb: 0.17	Tb: 0.46 (97%), 2.07 (3%)	>50

a Error on *ε* value is estimated ±5% on *ε* values and ±10% on *Φ*_Ln_ and *σ*_2P_. Error on *τ*_Ln_ is estimated ±0.03 ms.

b Energy of the excited triplet state is estimated from the wavelength at half-maximum on the onset of the time-gated phosphorescence spectrum of the Gd^3+^ analogue in PBS/glycerol 9 : 1 v/v recorded at 77 K.

c Extinction coefficient *ε* of dimers are estimated twice that of the monomer with the same antenna.

Excitation into these bands yields characteristic Tb^3+ 5^D_4_ → ^7^F_*J*_ (*J* = 6–0) or Eu^3+ 5^D_0_ → ^7^F_*J*_ (*J* = 0–4) emissions in the 450–750 nm range. The excitation spectra match the absorption spectra, which confirms sensitization of Ln^3+^ luminescence by antenna effect. Interestingly, with respect to quantum yields of Ln^3+^ emission, the antenna with the *ortho*-methyl group sensitizes Tb^3+^ better while the one without the *ortho*-methyl group sensitizes Eu^3+^ better.^36^ Quantum yields and lifetimes of Tb^3+^ emission decrease in the order L-^Me^Ar-OMe > L-^Me^Ar-NHAc > L-Ar-NHAc, following the T_1_ excited state energy (Table 1). Indeed, it seems that back-energy transfer from the Tb^3+ 5^D_4_ state to the antenna T_1_ state is responsible for this trend (the energy gap between antenna T_1_ and Tb^3+ 5^D_4_ states is particularly low for L-Ar-NHAc, *ca.* 1600 cm^−1^), which is confirmed by longer lifetimes in deoxygenated PBS (ESI†). For all these compounds, comparison of Ln^3+^ luminescence lifetimes measured in H_2_O and D_2_O indicates that the hydration number *q* is zero, in agreement with the saturated coordination sphere expected for these ligands (ESI†).^27,34^

**Fig. 3:**
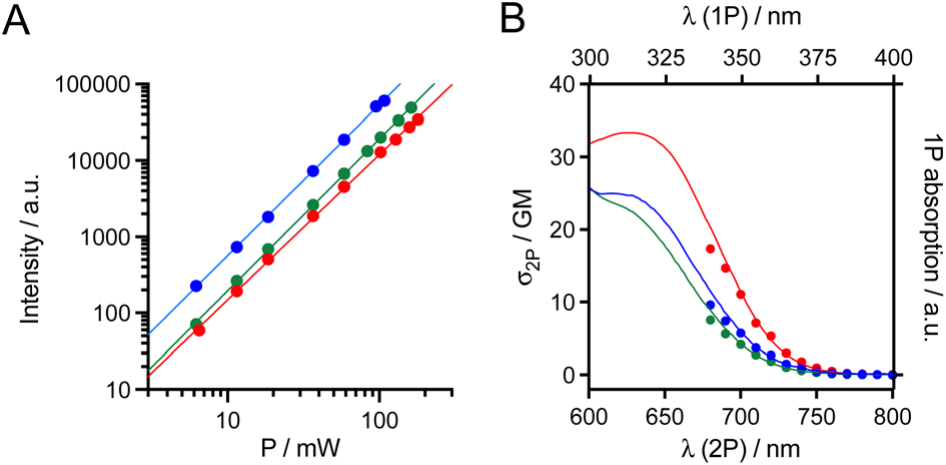
(A) Quadratic power dependance of the Ln^3+^ emission (*λ*_ex_ = 680 nm) for mTAT[Tb·L-^Me^Ar-OMe] (blue), mTAT[Tb L-^Me^Ar-NHAc] (green) and mTAT[Eu·L-Ar-NHAc] (red) in PBS. Data sets were fitted using *I* = *A* × *P*^*n*^ yielding *n* = 1.91, 1.99 and 1.98, respectively. (B) Superimposition of 2P absorption spectra (lower abscissa for wavelength, dots) and 1P absorption spectra (upper abscissa, solid lines) for mTAT[Tb·L-^Me^Ar-OMe] (blue), mTAT[Tb·L-^Me^Ar-NHAc] (green) and mTAT[Eu·L-Ar-NHAc] (red) in PBS.

The 2P absorption cross-sections (*σ*_2P_) in the NIR, between 680 and 800 nm, were determined for mTAT[Tb·L-^Me^Ar-OMe], mTAT[Tb·L-^Me^Ar-NHAc] and mTAT[Eu·L-Ar-NHAc] using the 2P excited fluorescence method (ESI†). For the three compounds, upon excitation at 720 nm, the characteristic Ln^3+^ emission is obtained and it displays a quadratic variation of intensity with respect to the incident laser power (Fig. 3A), the signature of a 2P-antenna effect. The 2P absorption spectrum was in good agreement with the wavelength-doubled 1P absorption one (Fig. 3B), indicating that the low-energy ILCT transition responsible for the sensitization of Ln^3+^ luminescence is one- and two-photon allowed. The values of *σ*_2P_ are low (Table 1), *ca.* 4–11 GM at 700 nm, and the compound with the freely rotating antenna, mTAT[Eu·L-Ar-NHAc], shows the highest cross-section. However, regarding the 2P brightness *B*_2P_ = *σ*_2P_ × *Φ*_Ln_, which quantifies the efficiency of the whole absorption/emission process, mTAT[Tb·L-^Me^Ar-OMe] appears to be the best compound with a *B*_2P_ value of 3.1 at 700 nm compared to 0.85 and 1.7 for mTAT[Tb·L-^Me^Ar-NHAc] and mTAT[Eu·L-Ar-NHAc], respectively. In cells, the main contributors to 2P-excited autofluorescence are NAD(P)H and FAD.^37,38^ They show emission bands with maxima at *ca.* 465 and 530 nm,^38^ respectively, and quantum yields of *ca.* 0.02 and 0.03 at physiological pH, respectively.^39^ Their 2P cross section under excitation around 700 nm is in the range 0.03–0.1 GM.^37^ Their 2P brightness is therefore lower than that of the Ln^3+^-based probes but the latter has to accumulate in sufficient amount in the cell to be detected efficiently, *i.e.* above the autofluorescence level.

Given the better luminescence properties of mTAT[Tb·L-^Me^Ar-OMe], mTAT[Tb·L-^Me^Ar-NHAc] and mTAT[Eu·L-Ar-NHAc] compared to the other three compounds, we have synthesized and characterized their dimeric analogues dTAT[Tb·L-^Me^Ar-OMe], dTAT[Tb·L-^Me^Ar-NHAc] and dTAT[Eu·L-Ar-NHAc] (ESI† and Table 1). Dimeric dTAT[Eu·L-Ar-NHAc] and monomeric mTAT[Eu·L-Ar-NHAc] exhibit identical photophysical properties (Table 1). However, for the Tb^3+^ compounds, the luminescence quantum yields and lifetimes of the dimeric compounds are lower than their monomeric counterparts, probably due to additional deactivation by the T_1_ state of the second Tb^3+^ complex of the dimer. However, should these compounds manage to reach the cell cytosol, the disulphide will be reduced due to its high glutathione content (*ca.* 4–5 mM).^40–42^ Consequently, the monomeric form is likely to better represent the species present inside cells with associated photophysical properties.

### Two-photon microscopy imaging of live cells

**Fig. 4:**
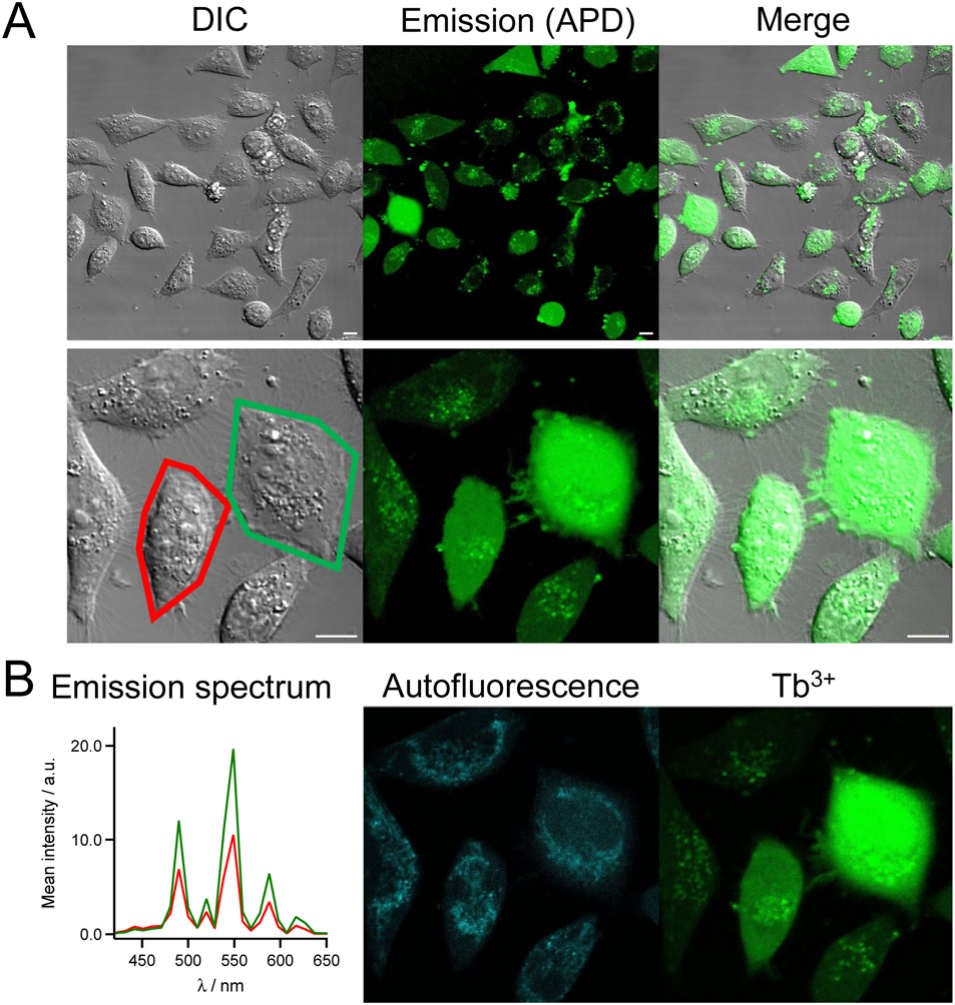
2PM imaging (*λ*_ex_ = 720 nm) of living HeLa cells incubated 1 h with dTAT[Tb·L-^Me^Ar-OMe] (10 μM) in RPMI medium. (A) Left panel: differential interference contrast (DIC) image; Middle panel: luminescence image recorded using a 420–650 nm bp filter and APD detection; Right panel: merge. Scale bars correspond to 10 μm. (B) Left panel: 2P-excited emission spectra (detected with PMT array) of cells outlined in red and green in panel A; Middle and right panels: autofluorescence and Tb^3+^ emission maps obtained by linear unmixing of 2P-excited spectral images recorded with the PMT.

The cell imaging properties of the dTAT[Ln·L] conjugates were investigated by microscopy using 2P excitation at 720 nm. Live HeLa cells were incubated for 1 h with the dTAT[Ln·L] conjugates then washed and imaged. Images obtained with dTAT[Tb·L-^Me^Ar-OMe] (10 μM) are shown in Fig. 4. In the DIC image (Fig. 4A, left), the cells phenotype is consistent with that of live cells. Luminescence emission under 2P excitation at 720 nm was collected with an avalanche photodiode (APD; Fig. 4A, middle panel) with a 420–650 nm bandpass (bp) filter. It was detected in all cells, but its intensity varied from cell to cell. Both punctate and diffuse emission signals are detected within cells, the latter covering in the entire cell. These features are reminiscent of the fluorescence pattern detected with dfTAT^32^ and, more generally, are characteristic of fluorophores internalized with CPP. A single APD does not allow to identify the emissive species based on its emission spectrum. Therefore, spectral detection was performed using a photomultiplier (PMT array of Quasar detectors). The left panel of Fig. 4B shows the emission spectra averaged within the entire cell arising from the two cells outlined in red and green in panel 4A, left. These emission spectra are dominated by the Tb^3+^ emission but a weaker contribution from autofluorescence background is also detected. Deconvolution of the luminescence images acquired in spectral mode resulted in the autofluorescence and Tb^3+^ emission maps showed in the middle and right panels of Fig. 4B, respectively. The autofluorescence localization is characteristic of the typical distribution of mitochondria, consistent with 2P excitation of NADH fluorescence. Tb^3+^ emission is detected in all cells, whether diffuse or punctate. The diffuse emission, which is observed in the entire cell, including nucleus, indicates successful cytosolic delivery of the Tb^3+^ complex, whereas the punctate emission may correspond to Tb^3+^ complexes still trapped in endosomes. After 4 h, the punctate distribution is still present. The luminescence decay of diffuse Tb^3+^ emission was measured in cells using TSLIM measurements^21^ and could be fitted bi-exponentially with lifetimes of 0.75 and 0.15 ms (Fig. S12†), which are both shorter than the lifetime measured in PBS solution with a spectrometer (Table 1). Nevertheless, these millisecond values confirm emission arising from the Tb^3+^ ion.

**Fig. 5:**
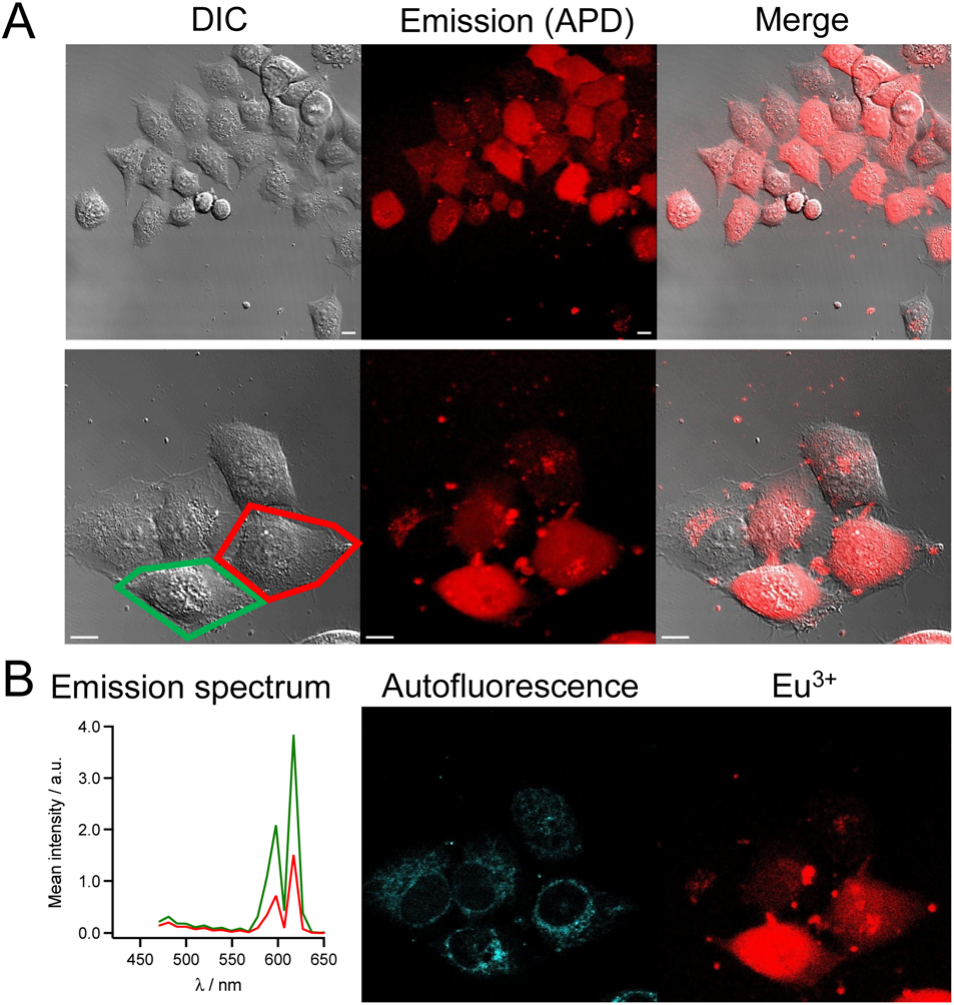
2PM imaging (*λ*_ex_ = 720 nm) of living HeLa cells incubated 1 h with dTAT[Eu·L-Ar-NHAc] (10 μM) in RPMI medium. (A) Left panel: DIC image; Middle panel: luminescence image recorded using a 465–680 nm bp filter and APD detection; Right panel: merge. Scale bars correspond to 10 μm. (B) Left panel: 2P-excited emission spectra (detected with PMT array) of cells outlined in red and green in panel A; Middle and right panels: autofluorescence and Eu^3+^ emission maps obtained by linear unmixing of 2P-excited spectral images recorded with the PMT.

Similar images and cell distributions were obtained with dTAT[Tb·L-^Me^Ar-NHAc] (Fig. S13†) and dTAT[Eu·L-Ar-HNAc] after 1 h incubation at 10 μM (Fig. 5). In the former case, Tb^3+^ emission was clearly detected in cells by spectral detection although it was weaker than for dTAT[Tb·L-^Me^Ar-OMe] compared to autofluorescence emission, consistent with the lower 2P brightness of the former. Nevertheless, the deconvolution of autofluorescence and Tb^3+^ emission (Fig. S13†) shows a Tb^3+^ signal that is both diffuse and punctate, with the diffuse component attesting to cytosolic delivery. A bi-exponential luminescence decay with lifetimes of 0.84 and 0.15 ms was measured in cells for the diffuse Tb^3+^ signal.

In the case of dTAT[Eu·L-Ar-HNAc], a large number of cells shows diffuse Eu^3+^ emission above the autofluorescence as shown in the spectrum in Fig. 5, with peaks at 590 and 615 nm. This confirms again the cytosolic delivery of the Eu^3+^ complex. Some cells show also punctate Eu^3+^ emission inside the cell but outside the nucleus, which may correspond to the Eu^3+^ probe trapped in endosomes or lysosomes. Small clumps of the Eu^3+^ probe are also observed in areas where no cell is present or sticked to the cell membrane. This may correspond to probe aggregates that form in the culture medium (not observed in drop of water solution of the probe, even at 100 μM). Within cells, the Eu^3+^ luminescence decay lifetime of diffuse emission was 0.65 ms. As for Tb^3+^ compounds, the lifetime measured in the living cell is shorter than in PBS solution. The lifetime measured in a drop of Ln^3+^ conjugate with the microscope is within margin error equal to the one determined in solution with a spectrometer, ruling out problems due to measurement procedure with the microscope. The reason is unclear but we can envisage that energy transfer from the excited Ln^3+^ to cell components (aromatic co-factors) happens. The reason of the bi-exponential decay of the Tb^3+^ needs further studies to be clarified.

**Fig. 6:**
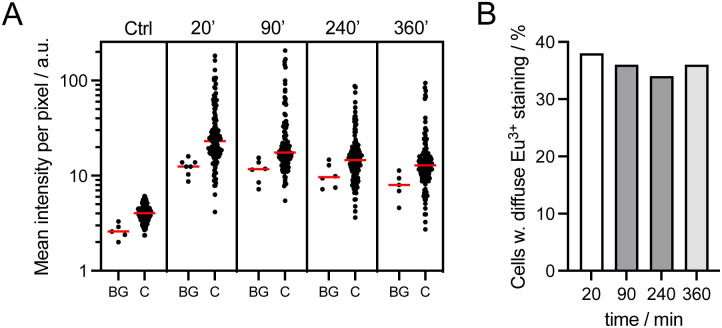
Time dependence of cell staining with dTAT[Eu·L-Ar-NHAc] (5 μM, 1 h). (A) Mean emission intensity measured in the red (590–680 bp filter) for background (BG) and within cells (C); median value is shown in the red. (B) Percentage of cells showing diffuse Eu^3+^ staining, as determined visually.

Cell staining with dTAT[Eu·L-Ar-HNAc] was also studied in a concentration dependent manner. At 2.5 μM (1 h incubation), cells only show punctate Eu^3+^ around the nucleus (Fig. S14†). Counter-staining with LysoView488™ (Biotium), a lysosome marker, shows excellent overlap with Eu^3+^ distribution (Fig. S15†), confirming that the punctate probe emission corresponds to endosomal/lysosomal entrapment. Next we examined the time dependence of the staining after 1 h incubation of HeLa cells with dTAT[Eu·L-Ar-HNAc] at 5 μM. The mean intensity of Eu^3+^ emission recorded using a 590–680 nm bp filter was measured within cells 20, 90, 240 and 360 min after washing (*ca.* 120 cells analysed at each time point) and compared to non-incubated HeLa cells as a control (Fig. 6A). The background emission was higher in incubated cells than in control, especially close to the surface. This may be attributed to dTAT[Eu·L-Ar-HNAc] adhered to the surface, as already described for CPP-rhodamine conjugates.^43^ The distribution of mean intensity within cells does not vary significantly with time up to 6 h. The number of cells showing cytosolic staining, *i.e.* diffuse Eu^3+^ emission was also determined. It remains constant (34–38%) over 6 hours after washing (Fig. 6B). Punctate Eu^3+^ emission trapped in endosomes is still observed after 6 h in some cells.

**Fig. 7:**
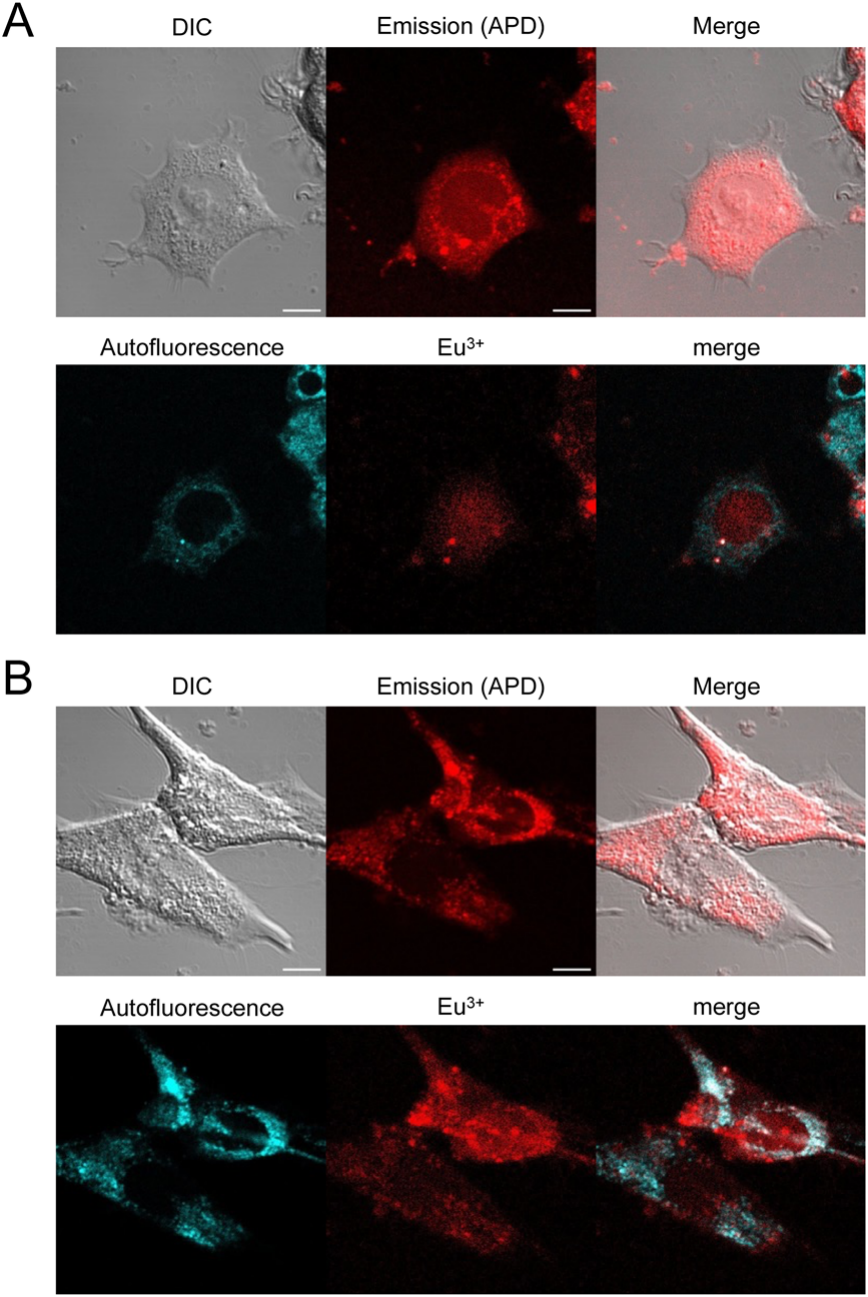
2PM imaging (*λ*_ex_ = 720 nm) of living HEK293T (A) and MRC5 (B) cells incubated 1 h with dTAT[Eu·L-Ar-NHAc] (10 μM) in RPMI medium. Top: DIC, emission recorded using using a 465–680 nm bp filter and merge; Bottom: linear unmixing of autofluorescence (AF) and Eu^3+^ emission and merge. Scale bars correspond to 10 μm.

Finally, internalization was confirmed in other cell types such as HEK293T or MRC5 cells with dTAT[Eu·L-Ar-HNAc] with successful cytosolic delivery demonstrated by the characteristic diffuse Eu^3+^ localization (Fig. 7). Interestingly, no significant internalization was observed with mTAT compounds in HeLa cells, demonstrating the importance of TAT dimerization for cell penetration efficiency and cytosolic delivery.

### Cytotoxicity

**Fig. 8:**
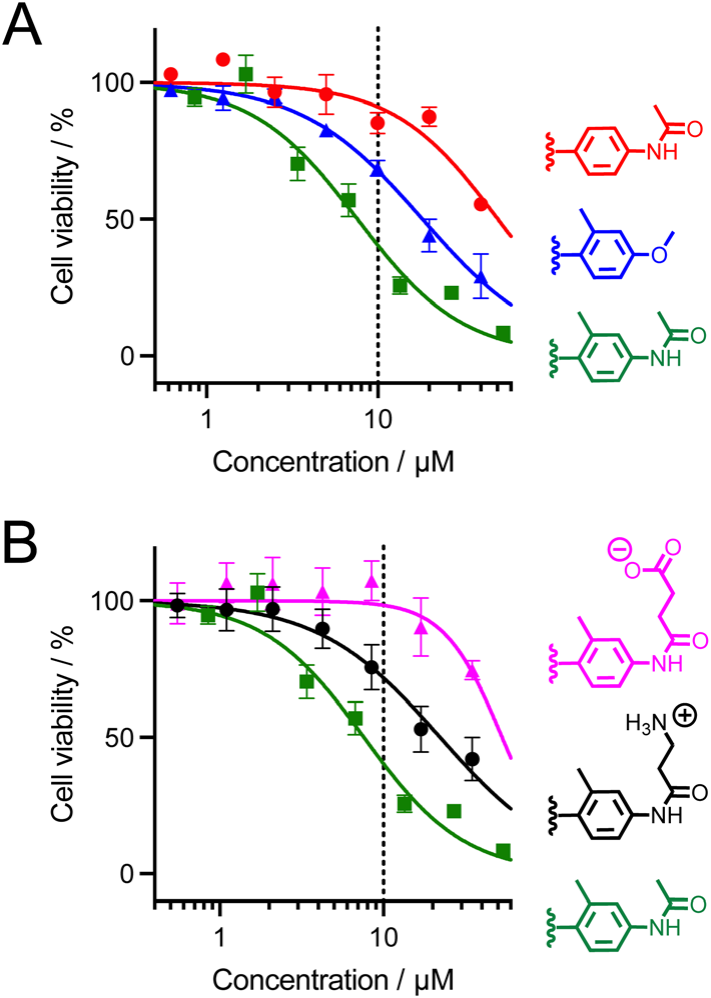
MTT proliferation assay for dTAT[Ln·L] conjugates. (A) Red: dTAT[Eu·L-Ar-NHAc]; blue: dTAT[Tb·L-^Me^Ar-OMe]; green: dTAT[Tb·L-^Me^Ar-NHAc]. (B) Pink: dTAT[Tb·L-^Me^Ar-NHSuc(−)]; black: dTAT[Tb·L-^Me^Ar-NHβAla(+)]; green: dTAT[Tb·L-^Me^Ar-NHAc]. Symbols correspond to experimental data and solid lines to the fit that yielded IC_50_ values given in Table 1. Error bars correspond to the SEM. The dotted line indicates the highest concentration used for 2P microscopy in this article (10 μM).

The cytotoxicity of dTAT[Ln·L] conjugates was evaluated using the MTT proliferation assay. The compound concentration causing a 50% reduction in proliferation (IC_50_) is given in Table 1 and plots of cell viability as a function of compound concentration are shown in Fig. 8 dTAT[Eu·L-Ar-HNAc] is the least toxic (IC_50_ > 50 μM), showing no toxicity at the 10 μM concentration used for 2PM. The IC_50_ of the two Tb^3+^ compounds carrying the *ortho*-methyl on the antenna are lower, with dTAT[Tb·L-^Me^Ar-HNAc] being the most toxic with an IC_50_ below 10 μM. We also observed stronger toxicity (IC_50_ = 2.5 μM) with a related compound bearing a carbazole instead of a methoxy or acetamide electrodonating group (this compound will be described in detail elsewhere). Suspecting that the toxicity might be related to the hydrophobicity of the antenna and its possible interaction with membrane lipids, we synthesized two analogues of the more toxic Tb^3+^ compound but with charged pendants on the amide electrodonating group: dTAT[Tb·L-^Me^Ar-NHβAla(+)] and dTAT[Tb·L-^Me^Ar-NHSuc(−)] with an additional positive and negative charge, respectively. Their luminescence properties (Table 1) are similar to those of their parent compound dTAT[Tb·L-^Me^Ar-NHAc], indicating that the introduction of the charge has little effect on the emission properties. Interestingly, however, the additional charge greatly reduces the toxicity (Table 1), increasing the IC_50_ values above 10 μM, which is the highest concentration used for 2P imaging experiments. It should be noted that the MTT assays were carried out with a 2 hour incubation instead of the 1 hour incubation for 2PM. Consequently, toxicity might be overestimated compared with 2PM conditions. 2PM imaging of HeLa cells showed that the additional charge does not alter the cell penetration properties: both dTAT[Tb·L-^Me^Ar-NHβAla(+)] and dTAT[Tb·L-^Me^Ar-NHSuc(−)] are efficiently delivered to the cytosol with characteristic diffuse emission (Fig. S16 and S17†). This demonstrates that the cytotoxicity of these compounds can be easily modulated by reducing the lipophilicity of the antenna, without affecting the photophysical, penetration and imaging properties.

### Toward multiplex imaging

**Fig. 9:**
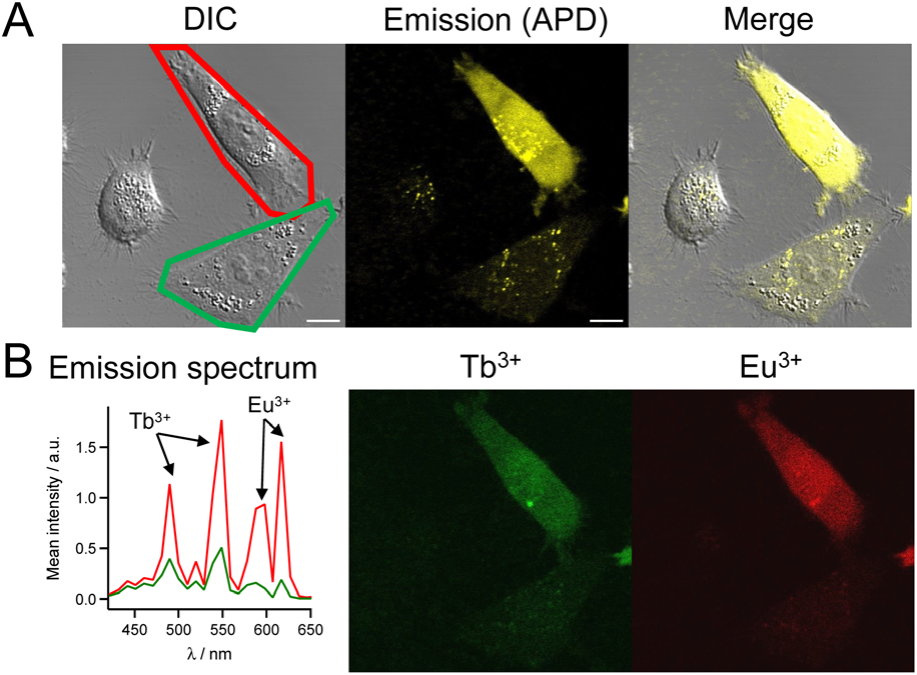
2PM imaging (*λ*_ex_ = 720 nm) of living HeLa cells incubated 1 h with dTAT[Tb·L-^Me^Ar-NHβAla(+)] (10 μM) and dTAT[Eu·L-Ar-NHAc] (3 μM) in RPMI medium. (A) Left panel: DIC image; Middle panel: luminescence image recorded using APD; Right panel: merge. Scale bars correspond to 10 μm. (B) Left panel: 2P-excited emission spectra (detection with PMT array) of cells outlined in red and green in panel A; Middle and right panels: Tb^3+^ and Eu^3+^ emission maps obtained by linear unmixing of 2P-excited spectral images recorded with the PMT.

Finally, since these compounds have similar 2P brightness, we attempted cell imaging with a Tb^3+^/Eu^3+^ mixture. HeLa cells were incubated for 1 h with dTAT[Tb·L-^Me^Ar-NHβAla(+)] (10 μM) and dTAT[Eu·L-Ar-NHAc] (5 μM), rinsed and imaged by 2P microscopy. A luminescence emission could be detected within the cells, the intensity of which again varied from cell to cell (Fig. 9). However, the diffuse emission was detected in many cells. The emission spectrum arising from cells under 2P excitation at 720 nm clearly shows fingerprint peaks of Tb^3+^ (490 and 540 nm) and Eu^3+^ (590 and 615 nm) emission (Fig. 9B). This indicates that both Tb^3+^ and Eu^3+^ complexes were co-internalized in the cells. Spectral deconvolution (Fig. 9B, right) showed that they were delivered to the cytosol because of the diffuse signal and co-localized well throughout the entire cell. A similar Tb^3+^/Eu^3+^ co-internalization could be obtained with dTAT[Tb·L-^Me^Ar-NHSuc(−)] (Fig. S18†). This opens the way to multiplex imaging with multiple Ln^3+^ probes in living cells.

## Conclusion

In the present work we report a family of luminescent lanthanide bioprobes, named dTAT[Ln·L], that are efficiently delivered into the cytosol of living cells and allow two-photon microscopy imaging. These probes are based on a TAT dimer functionalized by Ln^3+^ complexes, which exhibit 2P absorption properties thanks to a push–pull antenna. As a Ln^3+^ chelator (L), we choose DO3Apic but equipped with different π-extended picolinate moieties. It can be easily conjugated to peptides *via* the picolinate group and forms stable Tb^3+^ and Eu^3+^ complexes with good to excellent photophysical properties. Optical properties identified three candidates for 2PM imaging of cells, *i.e.* two Tb^3+^ and one Eu^3+^ complexes with quantum yields ≥15% and featuring 2P absorption. 2PM of live HeLa cells incubated for 1 h with these complexes conjugated to TAT dimers showed intense Ln^3+^ emission under 2P excitation at 720 nm despite their low 2P cross sections. Successful cytosolic delivery of the Ln^3+^ complex was observed in most cells with characteristic diffuse Tb^3+^ or Eu^3+^ emission within the entire cell. The conjugates can be used to image other cell types as demonstrated with HEK293T and MRC5 cells. Interestingly, we have shown that the potential toxicity of these conjugates can be easily reduced to acceptable levels by adding charges onto the antenna to reduce its lipophilicity. In contrast to the dimeric dTAT[Ln·L] compounds, the monomeric analogues mTAT[Ln·L] are unable to penetrate cells sufficiently for 2PM imaging. Hence, this article shows that the TAT dimer is a suitable scaffold for the design of Ln^3+^-based luminescent probes that have to be delivered into the cytosol of living cells.

The controlled delivery of dTAT[Ln·L] is really innovative compared to previous Ln^3+^ luminescent probes that required cell fixation to permeabilize the cell membranes for probe internalization or that accumulated in an uncontrolled manner in either lysosomes or mitochondria of living cells. Importantly, 2PM imaging with the dTAT[Ln·L] conjugates was performed using a commercial confocal microscope routinely used in biology laboratories and Ln^3+^ could be fully characterized inside cells by recording the emission spectrum and the millisecond luminescence decay lifetime.

Finally, given the design of these probes and the versatility offered by their synthetic pathway, there is room to improve their photophysical properties. The design described here opens the way to more sophisticated responsive probes with ratiometric detection based on emission intensity using mixtures of Ln^3+^ or emission lifetime.

## Data availability

The datasets supporting this article have been uploaded as part of the ESI.† They are also available from the authors on reasonable request.

## Author contributions

Conceptualization: O. S., O. M.; investigation: K. P. M., T. C., D. N., J.-H. C., L. B., B. C., G. M., S. E., V. M.-F., A. G., O. S.; validation: O. S., A. B., O. M., V. M.-F., A. G.; witting (original draft): O. S.; writing (review & editing): all authors. Visualization: O. S.

## Conflicts of interest

There are no conflicts to declare.

## Supplementary Material

SC-015-D4SC00896K-s001
