# Supplementary material for: Efficient cytosolic delivery of luminescent lanthanide bioprobes in live cells for two-photon microscopy
Source: Chem Sci. 2024 May 17;15(25):9694–702. doi: 10.1039/d4sc00896k (PMC11206396; doi:10.1039/d4sc00896k)
Supplement: SC-015-D4SC00896K-s001 [file SC-015-D4SC00896K-s001.pdf]

Electronic Supplementary Material (ESI) for Chemical Science.
This journal is © The Royal Society of Chemistry 2024

Efficient Cytosolic Delivery of Luminescent Lanthanide Bioprobes in Live Cells for Two-Photon Microscopy

Kyangwi P. Malikidogo,[a,b,+,#] Thibault Charnay,[a,b,+] Daouda Ndiaye,[a] Ji-Hyung Choi,[a] Lucile Bridou,[c] Baptiste Chartier,[a] Sule Erbek,[d,e] Guillaume Micouin,[c] Akos Banyasz,[c] Olivier Maury,[f] Véronique Martel-Frachet,[d,e] Alexei Grichine[d] and Olivier Sénèque*[a]

[a] Univ. Grenoble Alpes, CNRS, CEA, IRIG, LCBM (UMR 5249), F-38000 Grenoble, France.

[b] Univ. Grenoble Alpes, CNRS, DCM (UMR 5250), F-38000 Grenoble, France.

[c] Univ Lyon, ENS de Lyon, CNRS UMR 5182, Laboratoire de Chimie, Lyon F-69342, France.

[d] Univ. Grenoble Alpes, INSERM U1209, CNRS UMR 5309, Institute for Advanced Biosciences, F-38000 Grenoble, France.

[e] EPHE, PSL Research University, 4-14 rue Ferrus, 75014 Paris, France.

[+] These authors contributed equally to this work.

[#] Present address: Faculté des Sciences et Technologies, Université de Goma, B.P. 204 Goma, R. D. Congo.

Email: *olivier.seneque@cea.fr*

Supporting Information

Content

Abbreviations 2
Materials and methods 2
Synthesis 3
Peptide sequences 8
Peptide synthesis 9
1P spectroscopy 13
2P spectroscopy 15
2P microscopy 16
Cytotoxicity 21
References 22

Abbreviations

Alloc: allyloxycarbonyl; aq.: aqueous; Boc: *tert*-butyloxycarbonyl; DCC: dicyclohexylcarbodimide; DIEA: *N*,*N*-diisopropyl-ethylamine; DMAP: *N*,*N*-dimethylaminopyridine; dppf: 1,1'-*bis*(diphenylphosphino)ferrocene; EDTA, ethylenediamine-tetraacetic acid; ESI: electrospray ionization; Fmoc: 9-fluorenyl-methoxycarbonyl; HCTU: *O*-(1*H*-6-chlorobenzotriazole-1-yl)-1,1,3,3-tetramethyluronium hexafluorophosphate; HEPES: 2-(4-(2-hydroxyethyl)piperazin-1-yl)ethanesulfonic acid; HRMS: high resolution mass spectrometry; LRMS: low resolution mass spectrometry; MTT: 3-(4,5-dimethylthiazol-2-yl)-2,5-diphenyltetrazolium bromide ; NMP: *N*-methyl-2-pyrrolidone; Pbf: 2,2,4,6,7-pentameethyldihydrobenzofuran-5-sulfonyl; PyBOP: (benzotriazol-1-yloxy)tripyrrolidinophosphonium hexafluorophosphate; SPPS: solid-phase peptide synthesis; TIS: triisopropylsilane; Trt: trityl.

Materials and methods

Reagents and solvents: N-α-Fmoc-protected amino acids for peptide synthesis, HCTU and PyBOP coupling reagent and NovaPEG Rink Amide resin were purchased from Novabiochem or Iris Biotech. Other reagents for peptide synthesis, solvents, buffers and metal salts were purchased from Sigma-Aldrich. All buffer or metal solutions for spectroscopic measurements were prepared with ultrapure water produced by a Millipore Milli-Q purification system (purified to 18.2 MΩ.cm). Buffer solutions were treated with Chelex 100 resin (Bio-Rad) to remove trace metal ions.
HPLC purifications: Analytical HPLC/LRMS analyses were performed on an Agilent Infinity 1260 II system equipped with a 6125 MS (ESI) detector using a Waters XBridge BEH130 C18 (2.5 µm, 75 mm × 4.6 mm). Preparative HPLC separations were performed on a VWR LaPrepΣ system using Waters XBridge Peptide BEH130 C18 (5 µm, 150 mm × 19 mm) or Waters XBridge Peptide BEH130 C18 (5 µm, 150 mm × 10 mm) columns at flow rates of 14 or 6 mL/min, respectively. Mobile phase consisted in a gradient of solvent A (0.1 % TFA in H_2O) and B (0.1 % TFA in MeCN/H_2O 9:1). For analytical separations, Method B consisted in 5% B during 1 min followed by a 5 to 100 % B gradient in 13 min at 1 mL/min. Eluate was monitored by electronic absorption at 214, 280 and 331 nm as well as by LRMS (ESI+) detection.
NMR spectroscopy: ^{1}H, ^{13}C and DEPT NMR spectra were recorded at 400 MHz on a Varian Avance III 400 spectrometer at 298 K unless specified. Coupling constants (J) are measured in Hertz and are given with 0.5 Hz accuracy and the chemical shift (δ) are measured in ppm. All chemical shifts for ^{1}H and ^{13}C spectra were referenced to the residual solvent peak ($CDCl_3$ δ_H = 7.26 ppm and δ_C = 77.2 ppm; CD_3OD δ_H = 3.31 ppm and δ_C = 49.0 ppm). The following abbreviations were for peak multiplicities: s (singlet), d (doublet), t (triplet), m (multiplet), br (broad peak(s)).
Mass spectrometry: LRMS(ESI) analyses were performed on a Thermo Scientific LXQ spectrometer. HRMS (ESI) were performed on a Thermo Scientific LTQ Orbitrap XL spectrometer or on a Waters Xevo G2-S QTof spectrometer with electrospray ionization.
Optical spectroscopy: UV-Vis absorption spectra were recorded on a Perkin-Elmer Lambda 35 spectrophotometer or on a Varian Cary 50 spectrometer, both equipped with a thermo-regulated cell holder. Luminescence spectra were measured on a Varian Cary Eclipse spectrometer equipped with a thermo-regulated cell holder or on a modular Fluorolog FL3-22 spectrometer from Horiba-Jobin Yvon-Spex equipped with a double-grating excitation monochromator and an iHR320 imaging spectrometer coupled to Hamamatsu R928P and Hamamatsu R5509 photomultipliers for visible and NIR detection, respectively. Emission spectra were corrected for wavelength-dependent detector response. Time-gated Ln^{3+} luminescence spectra were acquired with 100 µs time delay and 2 ms gate time on the Varian Cary Eclipse spectrometer. Ln^{3+} luminescence lifetimes were measured using the Varian Cary Eclipse spectrometer.

Synthesis

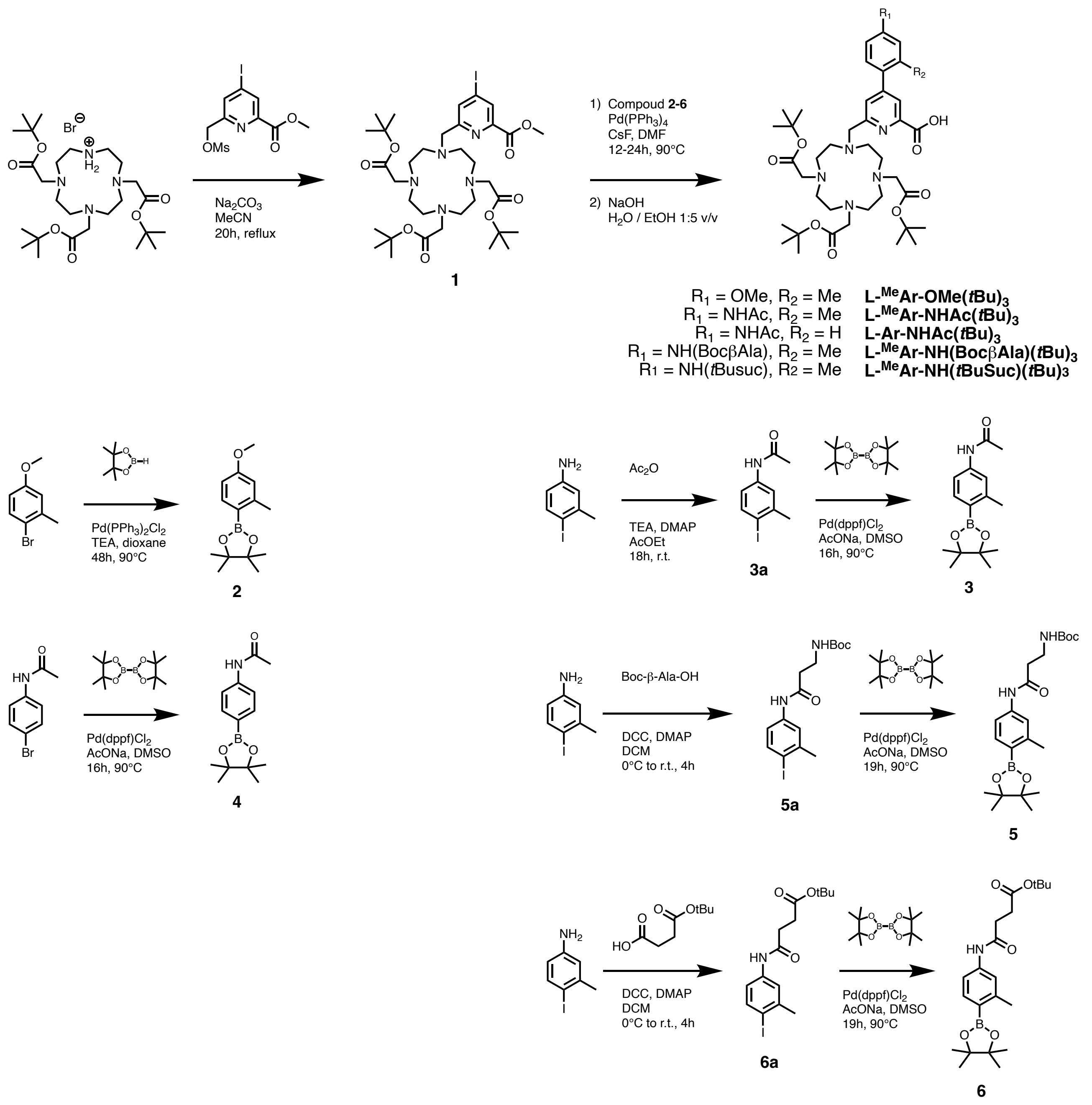

Fig. S1 Synthetic pathways for pro-ligands L(tBu)3.

Compound 1: Na_2CO_3 (1.58 g, 15.0 mmol) was added to a solution of [1,4,7-*tris*(*tert*-butoxycarbonylmethyl)]-1,4,7,10-tetraazacyclododecane hydrobromide[1] (1.8 g, 3.0 mmol) in MeCN (10 mL). Then a solution of methyl 4-iodo-6-(((methylsulfonyl)oxy)methyl)picolinate[2] (1.1 g, 3.0 mmol) in MeCN (10 mL) was added. The mixture was heated at reflux for 20 h, then cooled to room temperature and filtrated over Celite. The solvent was evaporated under reduced pressure and the residue was triturated in AcOEt and sonicated to give **1** as a white powder (1.92 g, 82 %). ^{1}H NMR (400 MHz, $CDCl_3$): δ = 8.32 (d, J = 1.5, 1H), 7.96 (d, J = 1.5, 1H), 3.90 (s, 3H), 3.76 (br, 2H), 3.40-2.20, (m, 22H), 1.46 (s, 9H), 1.34 (s, 18H); $^{13}C\{^{1}H\}$ NMR (100 MHz, $CDCl_3$): δ = 172.4, 171.6, 164.1, 160.1, 147.0, 136.2, 132.9, 107.5, 82.3, 82.1, 58.8, 56.3, 56.1, 53.2, 50.7, 28.1, 28.0; LRMS (ESI+): monoisotopic m/z = 790.3 (+) / calculated m/z = 790.3 $[M+H]^+$ for M = $C_{34}H_{56}N_5O_8I$; HRMS (ESI+): monoisotopic m/z = 790.3239 (+) / calculated m/z = 790.3246 $[M+H]^+$ for M = $C_{34}H_{56}N_5O_8I$.

4-(4,4,5,5-tetramethyl-1,3,2-dioxaborolan-2-yl)-3-methylanisole 2: To a solution of 4-bromo-3-methylanisole (157 µL, 1.1 mmol) and 4,4,5,5-tetramethyl-1,3,2-dioxa-borolane (260 µL, 1.8 mmol) in dry dioxane (15 mL) were added *bis*(triphenylphosphine)palladium(II) dichloride (25 mg, 0.03 mmol) and triethylamine (500 µL, 3.6 mmol). The reaction was stirred at 90°C under argon for 48 h, then cooled to room temperature and filtrated over Celite. The solvent was removed under reduced pressure and the solid obtained was dissolved in DCM. The organic phase was washed with water twice. The organic layer was dried over Na_2SO_4 and the solvent was removed under reduced pressure. The crude product was purified by flash chromatography (silica gel, Cyclohexane/DCM 1:1) to give a white powder (195 mg, 88 %). ^1H NMR (400 MHz, $CDCl_3$): δ = 7.72 (d, J = 8.5, 1H), 6.71 (m, 2H), 3.81 (s, 3H), 2.53 (s, 3H), 1.33 (s, 12H); $^{13}C\{^1H\}$ NMR (100 MHz, $CDCl_3$): δ = 161.8, 147.4, 138.0, 115.7, 110.3, 83.3, 55.1, 25.0, 22.5. Spectroscopic data corresponded to literature.[3]

L-^{Me}Ar-OMe(*t*Bu)$_3$: Compound **2** (61 mg, 0.25 mmol) and compound **1** (147 mg, 0.19 mmol) were dissolved in anhydrous DMF (5 mL). Cesium fluoride (109 mg, 0.72 mmol) and *tetrakis*(triphenylphosphine)palladium(0) (28 mg, 0.025 mmol, 0.1 eq.) were added. The reaction was stirred at 90 °C for 24 h. The reaction mixture was cooled to room temperature and the solvent was removed under reduced pressure. The residue was dissolved in DCM and the solution was washed with water. The organic layer was dried over Na_2SO_4 and the solvent was evaporated. The crude product was dissolved in EtOH (20 mL). NaOH 6 M (4 mL) was added dropwise and the reaction mixture was stirred for 20 min. The solution was neutralized using saturated $NaHCO_3$. After extraction with AcOEt, the organic layer was washed with water and dried over Na_2SO_4. The solvent was removed under reduced pressure. The crude product was purified by HPLC to give a yellow powder (98 mg, 46 % calculated based of the formula **L-^{Me}Ar-OMe(*t*Bu)$_3$·3TFA**). HPLC (anal.): t_R = 10.9 min (method B); ^1H NMR (400 MHz, $CDCl_3$): δ = 8.12 (s, 1H), 7.50 (s, 1H), 7.15 (d, J = 9.0, 1H), 6.82 (m, 2H), 4.51 (s, 2H), 3.88 (s, 2H), 3.84 (s, 3H), 3.7–3.1 (m, 20H), 2.28 (s, 3H) 1.43 (s, 9H), 1.38 (s, 18H); $^{13}C\{^1H\}$ NMR (100 MHz, $CDCl_3$): δ = 168.3, 167.3, 166.0 161.2 (q, J = 37, TFA), 160.4, 153.2, 148.0, 136.8, 131.0, 129.8, 127.4, 125.8, 116.6, 116.3 (q, J = 291, TFA), 112.1, 84.3, 83.6, 57.6, 55.5, 55.3, 54.6, 50.9, 50.6, 49.5, 28,1, 28.0, 20.7; LRMS (ESI+): monoisotopic m/z = 770.7 (+), 792.7 (+) / calculated m/z = 770.5 $[M+H]^+$, 792.7 $[M+Na]^+$, for M = $C_{41}H_{63}N_5O_9$; HRMS (ESI+): monoisotopic m/z = 770.4703 (+) / calculated m/z = 770.4699 $[M+H]^+$ for M = $C_{41}H_{63}N_5O_9$.

4-(4,4,5,5-tetramethyl-1,3,2-dioxaborolan-2-yl)-3-methylacetanilide (3): Compound **3a**[4] (300 mg, 1.1 mmol), bis(pinacolato)diboron (305 mg, 1.2 mmol), [1,1'-*bis*(diphenylphosphino)ferrocene]palladium(II) dichloride (40 mg, 0.05 mmol) and sodium acetate (214 mg, 2.2 mmol) were dissolved in dried DMSO (2 mL). The solution was degassed using argon during 30 min. The reaction was stirred at 90 °C under argon for 16 h. The reaction mixture was cooled to room temperature then it was diluted in AcOEt and filtrated over Celite. The organic layer was washed with saturated NH_4Cl twice and then with water. The organic layer was dried over Na_2SO_4 and the solvent was removed under reduced pressure. The crude product was purified by flash chromatography (silica gel, DCM/MeOH 98:2) to give a colourless oil (275 mg, 91 %).^1H NMR (400 MHz, $CDCl_3$): δ = 7.71 (d, J = 8.0, 1H), 7.33 (s, 1H), 7.29 (d, J = 8.0, 1H), 2.52 (s, 3H), 2.16 (s, 3H), 1.33 (s, 12H); $^{13}C\{^1H\}$ NMR (100 MHz, $CDCl_3$): δ = 168.4, 146.6, 140.3, 137.3, 120.5, 115.8, 83.6, 53.7, 25.0, 22.5; LRMS (ESI+): monoisotopic m/z = 276.2 (+) / calculated m/z = 276.2 $[M+H]^+$, for M = $C_{15}H_{23}NO_3B$.

L-^{Me}Ar-NHAc(*t*Bu)$_3$: Compound **3b** (60 mg, 0.23 mmol), compound **1** (160 mg, 0.2 mmol), cesium fluoride (90 mg, 0.59 mmol) and *tetrakis*(triphenylphosphine)palladium(0) (20 mg, 0.02 mmol) were dissolved in anhydrous DMF (2.5 mL). The reaction was stirred during 12 h at 90 °C under argon. The reaction mixture was cooled to room temperature and it was diluted using AcOEt. The solution was washed with saturated $NaHCO_3$ then water. The organic layer was dried over Na_2SO_4 and the solvent was removed under reduced pressure. The crude product was dissolved in EtOH (20 mL). NaOH 6 M (4 mL) was added dropwise and the reaction mixture was stirred for 10 min at room temperature. The solution was neutralized by dropwise addition of HCl 6 M (*ca.* 4 mL) and a precipitate was formed. The suspension was centrifugated and AcOEt was added to the supernatant. The organic layer was washed with water, dried over Na_2SO_4 and

the solvent was removed under reduced pressure. The product obtained was purified by HPLC to give a white powder (84 mg, 38 % calculated based of the formula **L-^{Me}Ar-NHAc (*t*Bu)$_3$·3TFA**). HPLC (anal.): t_R = 9.7 min (method B); ^{1}H NMR (400 MHz, CD_3OD): δ = 8.15 (s, 1H), 7.72 (d, J = 1.0, 1H), 7.57 (m, 2H), 7.24 (d, J = 8.0, 1H), 4.66 (s, 2H), 3.99-3.12 (br, 22H), 2.34 (s, 3H), 2.15 (s, 3H), 1.49 (s, 9H), 1.34 (s, 18H); ^{13}C{^{1}H} NMR (100 MHz, CD_3OD): δ = 171.9, 167.4, 162.5 (q, J = 38, TFA), 153.3, 149.0, 141.0, 137.1, 134.3, 131.2, 129.4, 126.8, 123.1, 119.5, 119.0, 118.2 (q, J = 291, TFA), 85.4, 84.0, 58.1, 56.0, 55.2, 52.2, 50.7, 28.4, 23.9, 20.8; LRMS (ESI+): monoisotopic *m/z* = 797.5 (+) / calculated *m/z* = 797.5 $[M+H]^+$, for M = $C_{42}H_{64}N_6O_9$; HRMS (ESI+): monoisotopic *m/z* = 797.4809 (+) / calculated *m/z* = 797.4808 $[M+H]^+$ for M = $C_{42}H_{64}N_6O_9$.

(4-(4,4,5,5-tetramethyl-1,3,2-dioxaborolan-2-yl)acetanilide (4): 4-bromoacetanilide (300 mg, 1.4 mmol), bis(pinacolato)diboron (391 mg, 1.5 mmol), [1,1'-*bis*(diphenylphosphino)ferrocene]palladium(II) dichloride (102 mg, 0.1 mmol) and sodium acetate (275 mg, 2.8 mmol) were dissolved in DMSO (2 mL). The solution was degassed using argon during 30 min. The reaction was stirred during 24 h at 90 °C under argon. The reaction mixture was diluted in AcOEt. The organic layer was washed with saturated NaCl then water and dried over Na_2SO_4. The solvent was removed under reduced pressure. The crude product was purified by flash chromatography (silica gel, AcOEt/Cyclohexane 30:70) to give a yellow oil (284 mg, 78 %). ^{1}H NMR (400 MHz, $CDCl_3$): δ = 7.76 (d, J = 8.5, 2H), 7.50 (d, J = 8.5, 2H), 7.16 (s, 1H), 2.19 (s, 3H), 1.35 (s, 12H); ^{13}C{^{1}H} NMR (100 MHz, $CDCl_3$): δ = 168.4, 140.6, 136.0, 118.6, 83.9, 29.9, 25.0. Spectroscopic data correspond to literature.[5]

L-Ar-NHAc(*t*Bu)$_3$: Compound **4** (68 mg, 0.26 mmol), compound **1** (133 mg, 0.17 mmol), cesium fluoride (83 mg, 0.55 mmol) and *tetrakis*(triphenylphosphine)palladium(0), polymer-bound (25 mg, 0.01 mmol) were dissolved in anhydrous DMF (2.5 mL). The reaction was stirred at 90 °C under argon for 24 h. The reaction mixture was cooled to room temperature and filtered over Celite. The solvent was removed under reduced pressure. The residue was dissolved in AcOEt. The organic phase was washed with saturated $NaHCO_3$ then water and dried over Na_2SO_4. The solvent was removed under reduced pressure. The crude product was dissolved in EtOH (20 mL). NaOH 6 M (4 mL) was added dropwise and the reaction mixture was stirred for 10 min at room temperature. The solution was neutralized by dropwise addition of HCl 6 M (*ca.* 4 mL) and a precipitate was formed. The solution was centrifugated and AcOEt was added to the supernatant. The organic phase was washed with water. The organic layer was dried over Na_2SO_4 and the solvent was removed under reduced pressure. The product was purified by HPLC to give a white powder (88 mg, 48 % calculated based of the formula **L-Ar-NHAc(*t*Bu)$_3$·3TFA**). HPLC (anal.): t_R = 9.4 min (method B); ^{1}H NMR (400 MHz, CD_3OD): δ = 8.41 (s, 1H), 8.01 (d, J = 1.5, 1H), 7.76 (s, 4H), 4.67 (s, 2H), 4.20-3.10 (m, 22H), 2.17 (s, 3H), 1.48 (s, 9H), 1.32 (s, 18H); ^{13}C{^{1}H} NMR (100 MHz, CD_3OD): δ = 171.9, 167.4, 162.5 (q, J = 38, TFA), 151.6, 149.8, 142.1, 132.7, 128.8, 126.1, 123.3, 121.4, 118.0 (q, J = 291, TFA), 85.4, 84.2, 58.4, 56.0, 55.2, 52.6, 50.7, 28.4, 24.0; HRMS (ESI+): monoisotopic *m/z* = 783.4655 (+) / calculated *m/z* = 783.4651 $[M+H]^+$ for M = $C_{41}H_{62}N_6O_9$.

***tert*-butyl(3-((4-iodo-3-methylphenyl)amino)-3-oxopropyl)carbamate (5a):** 4-iodo-3-methylaniline (595 mg, 2.6 mmol) was dissolved in DCM (5 mL) with Boc-β-Ala-OH (478 mg, 2.5 mmol) and DMAP (16.5 mg, 0.2 mmol). The solution was stirred for 10 min at 0 °C; then DCC (524 mg, 2.5 mmol) was added and a precipitate was formed. The solution was stirred at 0 °C to room temperature for 4 h. The reaction mixture was diluted in DCM and filtered. The organic phase was washed using a saturated $NaHCO_3$ then water. The organic layer was dried over Na_2SO_4 and the solvent was removed under reduced pressure. The crude product was purified by flash chromatography (silica gel, AcOEt/cyclohexane 20:80) to give a colourless powder (810 mg, 79 %). ^{1}H NMR (400 MHz, $CDCl_3$): δ = 7.82 (br, 1H), 7.70 (d, J = 8.5, 1H), 7.49 (s, 1H), 7.09 (d, J = 8.5, 1H), 5.13 (b, 1H), 3.48 (m), 2.58 (t, J = 11.5, 2H), 2.39 (s, 3H), 1.43 (s, 9H); ^{13}C{^{1}H} NMR (100 MHz, $CDCl_3$): δ = 169.7, 142.1, 139.2, 138.2, 121.0, 118.9, 94.4, 79.8, 37.8, 36.4, 28.4, 28.2; LRMS (ESI+): monoisotopic *m/z* = 405.0 (+), 427.0 (+) / calculated *m/z* = 405.0 $[M+H]^+$, 427.0 $[M+Na]^+$ for M = $C_{15}H_{21}N_2O_3I$.

***tert*-butyl(3-((3-methyl-4-(4,4,5,5-tetramethyl-1,3,2-dioxaborolan-2-yl)phenyl)amino)-3-oxopropyl)carbamate (5)**: Compound **5a** (463 mg, 1.1 mmol), *bis*(pinacolato)diboron (305 mg, 1.2 mmol), [1,1'-*bis*(diphenylphosphino)-ferrocene]palladium(II) dichloride (40 mg, 0.05 mmol) and sodium acetate (215 mg, 2.6 mmol) were dissolved in dried DMSO (2 mL). The solution was degassed using argon during 45 min. The reaction was stirred at 90 °C under argon for 18 h then cooled to room temperature. The reaction mixture was diluted in AcOEt and filtrated over Celite. The organic layer was washed with saturated NH_4Cl twice and then with water. The organic layer was dried over Na_2SO_4 and the solvent was removed under reduced pressure. The crude product was purified by flash chromatography (silica gel, AcOEt/cyclohexane 10:90) to give a slightly yellow oil (263 mg, 59 %). ^{1}H NMR (400 MHz, $CDCl_3$): δ = 7.74 (d, J = 8.0, 1H), 7.59 (br, 1H), 7.39 (s, 1H), 7.34 (d, J = 8.0, 1H), 5.19 (b, 1H), 3.51 (m, 2H), 2.62 (t, J = 11.5, 2H), 2.54 (s, 3H), 1.46 (s, 9H), 1.36 (s, 12H); ^{13}C{^{1}H} NMR (100 MHz, $CDCl_3$): δ = 182.0, 167.7, 146.4, 139.9, 137.1, 120.4, 115.6, 83.4, 37.7, 36.5, 28.4, 28.4, 24.9, 22.3; LRMS (ESI+): monoisotopic *m/z* = 405.0 (+) / calculated *m/z* = 405.3 $[M+H]^+$ for M = $C_{21}H_{33}N_2O_5B$.

L-^{Me}Ar-NH(BocβAla)(*t*Bu)$_3$: Compound **5** (187 mg, 0.44 mmol), compound **1** (300 mg, 0.38 mmol), cesium fluoride (173 mg, 1.14 mmol) and *tetrakis*(triphenylphosphine)palladium(0) (95 mg, 0.038 mmol) were dissolved in anhydrous DMF (3 mL). The reaction was stirred at 90 °C under argon for 20 h then cooled to room temperature. The reaction mixture was diluted using AcOEt and filtrated over Celite. The solution was extracted with saturated $NaHCO_3$ then water. The organic layer was dried over Na_2SO_4 and the solvent was removed under reduced pressure. The crude product was dissolved in EtOH (20 mL). NaOH 6 M (4 mL) was added dropwise and the reaction mixture was stirred for 10 min at room temperature. The solution was neutralized by dropwise addition of HCl 6 M (*ca.* 4 mL) and a precipitate was formed. The suspension was centrifugated and the supernatant was removed under reduced and the residue was solubilized in AcOEt. The solution was washed with water, dried over Na_2SO_4 and the solvent was removed under reduced pressure. The product obtained was purified by HPLC to give a white powder (259 mg, 49 % calculated based of the formula **L-Ar-NH(BocβAla)(*t*Bu)$_3$·4TFA**). HPLC (anal.): t_R = 10.7 min (method B); ^{1}H NMR (400 MHz, CD_3OD): δ = 8.17 (s, 1H), 7.74 (d, J = 1.0, 1H), 7.62 (s, 1H), 7.60 (s, 1H), 7.26 (d, J = 8.5, 1H), 4.69 (s, 2H), 4.27-3.47 (br, 18H), 3.43 (t, J = 7.0, 2H), 3.31-2.92 (br, 6H), 2.60 (t, J = 7.0, 2H), 2.36 (s, 3H), 1.51 (s, 9H), 1.45 (s, 9H), 1.36 (s, 18H); ^{13}C{^{1}H} NMR (100 MHz, CD_3OD): δ = 172.5, 167.4, 162.2 (q, J = 38, TFA), 153.2, 149.0, 140.9, 137.1, 134.2, 131.2, 129.4, 126.8, 123.2, 117.7 (q, J = 291, TFA), 116.3, 84.0, 80.2, 58.1, 56.0, 55.1, 52.5, 50.6, 38.3, 37.9, 28.7, 28.4, 20.8; LRMS (ESI+): monoisotopic *m/z* = 926.5 (+) / calculated *m/z* = 926.6 $[M+H]^+$, for M = $C_{48}H_{75}N_7O_{11}$; HRMS (ESI+): monoisotopic *m/z* = 926.5581 (+) / calculated *m/z* = 926.5597 $[M+H]^+$ for M = $C_{48}H_{75}N_7O_{11}$.

***tert*-butyl 4-((4-iodo-3-methylphenyl)amino)-4-oxobutanoate (6a) :** 4-iodo-3-methylaniline (595 mg, 2.6 mmol) was dissolved in DCM (5 mL) with 4-(tert-butoxy)-4-oxobutanoic acid (436 mg, 2.5 mmol) and DMAP (16.5 mg, 0.2 mmol). The solution was stirred for 10 min at 0 °C; then DCC (524 mg, 2.5 mmol) was added and a precipitate was formed. The solution was stirred at 0 °C to room temperature for 4 h. The reaction mixture was diluted in DCM and filtered. The organic phase was washed using a saturated $NaHCO_3$ then water. The organic layer was dried over Na_2SO_4 and the solvent was removed under reduced pressure. The crude product was purified by flash chromatography (silica gel, AcOEt/cyclohexane 30:70) to give a colourless oil (732 mg, 75 %). ^{1}H NMR (400 MHz, $CDCl_3$): δ = 7.89 (br, 1H), 7.69 (d, J = 8.5, 1H), 7.47 (s, 1H), 7.07 (d, J = 8.5, 1H), 2.72-2.57 (m, 4H), 2.40 (s, 3H), 1.48 (s, 9H); ^{13}C{^{1}H} NMR (100 MHz, $CDCl_3$): δ = 172.7, 170.1, 142.0, 139.1, 138.2, 120.9, 118.8, 94.1, 81.4, 34.0, 32.5, 30.8, 28.1; LRMS (ESI+): monoisotopic *m/z* = 390.0 (+), 412.0 (+) / calculated *m/z* = 390.0 $[M+H]^+$, 412.0 $[M+Na]^+$ for M = $C_{15}H_{20}NO_3I$.

***tert*-butyl 4-((3-methyl-4-(4,4,5,5-tetramethyl-1,3,2-dioxaborolan-2-yl)phenyl)amino)-4-oxobutanoate (6)**: Compound **6a** (446 mg, 1.1 mmol), *bis*(pinacolato)diboron (307 mg, 1.2 mmol), [1,1'-*bis*(diphenylphosphino)-ferrocene]palladium(II) dichloride (41 mg, 0.05 mmol) and sodium acetate (237 mg, 2.9 mmol) were dissolved in dried

DMSO (2 mL). The solution was degassed using argon during 45 min. The reaction was stirred during 18 h at 90 °C under argon. The reaction mixture was diluted in AcOEt and filtrated over Celite. The organic layer was washed with saturated NH_4Cl twice and then with water. The organic layer was dried over Na_2SO_4 and the solvent was removed under reduced pressure. The crude product was purified by flash chromatography (silica gel, AcOEt/cyclohexane 20:80) to give a slightly yellow oil (269 mg, 60 %). ^{1}H NMR (400 MHz, $CDCl_3$): δ = 7.72 (d, J = 8.0, 1H), 7.36 (s, 1H), 7.32 (d, J = 8.0, 1H), 2.70-2.59 (m, 4H), 2.53 (s, 3H), 1.47 (s, 9H), 1.35 (s, 12H); $^{13}C\{^1H\}$ NMR (100 MHz, $CDCl_3$): δ = 172.6, 170.1, 146.3, 140.2, 137.0, 120.4, 115.6, 83.3, 81.2, 32.6, 30.9, 30.8, 28.1, 24.9, 22.3; LRMS (ESI+): monoisotopic *m/z* = 390.0 (+) / calculated *m/z* = 390.2 $[M+H]^+$, for M = $C_{21}H_{32}NO_5B$.

L-^{Me}Ar-NH(*t*BuSuc)(*t*Bu)$_3$: Compound **6** (185 mg, 0.48 mmol), compound **1** (300 mg, 0.38 mmol), cesium fluoride (173 mg, 1.14 mmol) and *tetrakis*(triphenylphosphine)palladium(0) (95 mg, 0.038 mmol) were dissolved in anhydrous DMF (3 mL). The reaction was stirred at 90 °C under argon for 20 h then cooled to room temperature. The reaction mixture was diluted using AcOEt and filtrated over Celite. The solution was extracted with saturated $NaHCO_3$ then water. The organic layer was dried over Na_2SO_4 and the solvent was removed under reduced pressure. The crude product was dissolved in EtOH (20 mL). NaOH 6 M (4 mL) was added dropwise and the reaction mixture was stirred for 10 min at room temperature. The solution was neutralized by dropwise addition of HCl 6 M (*ca.* 4 mL) and a precipitate was formed. The suspension was centrifugated and the supernatant was removed under reduced and the residue was solubilized in AcOEt. The solution was washed with water, dried over Na_2SO_4 and the solvent was removed under reduced pressure. The product obtained was purified by HPLC to give a white powder (266 mg, 56 % calculated based of the formula **L-^{Me}Ar-NH(*t*BuSuc)(*t*Bu)$_3$·3TFA**). HPLC (anal.): t_R = 9.3 min (method B); ^{1}H NMR (400 MHz, CD_3OD): δ = 8.17 (s, 1H), 7.75 (d, J = 1.0, 1H), 7.61 (s, 1H), 7.59 (s, 1H), 7.26 (d, J = 8.5, 1H), 4.70 (s, 2H), 4.39-2.89 (br, 22H), 2.67 (m, 4H), 2.36 (s, 3H), 1.51 (s, 9H), 1.47 (s, 9H), 1.35 (s, 18H); $^{13}C\{^1H\}$ NMR (100 MHz, CD_3OD): δ = 173.6, 173.0, 162.2 (q, J = 38, TFA), 153.2, 149.0, 142.9, 141.0, 137.1, 134.1, 131.2, 129.5, 126.7, 123.0, 119.0 (q, J = 291, TFA), 116.3, 84.0, 81.8, 58.1, 56.0, 55.1, 52.5, 50.6, 32.5, 31.4, 28.4, 28.3, 20.8; LRMS (ESI+): monoisotopic *m/z* = 911.5 (+) / calculated *m/z* = 911.5 $[M+H]^+$, for M = $C_{48}H_{74}N_6O_{11}$; HRMS (ESI+): monoisotopic *m/z* = 911.5464 (+) / calculated *m/z* = 911.5488 $[M+H]^+$ for M = $C_{48}H_{74}N_6O_{11}$.

Peptide sequences

AcK-TAT-RA resin: Ac-Lys-Arg(Pbf)-Lys(Boc)-Lys(Boc)-Arg(Pbf)-Arg(Pbf)-Gln(Trt)-Arg(Pbf)-Arg(Pbf)-Arg(Pbf)-Gly-Rink Amide resin
CK-TAT-RA resin: Boc-Cys(Trt)-Lys-Arg(Pbf)-Lys(Boc)-Lys(Boc)-Arg(Pbf)-Arg(Pbf)-Gln(Trt)-Arg(Pbf)-Arg(Pbf)-Arg(Pbf)-Gly-Rink Amide resin

mTAT[L-^{Me}Ar-OMe]: Ac-K(L-^{Me}Ar-OMe)RKKRRQRRRG-NH_2
mTAT[Eu·L-^{Me}Ar-OMe]: Ac-K(Eu·L-^{Me}Ar-OMe)RKKRRQRRRG-NH_2
mTAT[Tb·L-^{Me}Ar-OMe]: Ac-K(Tb·L-^{Me}Ar-OMe)RKKRRQRRRG-NH_2
mTAT[Gd·L-^{Me}Ar-OMe]: Ac-K(Gd·L-^{Me}Ar-OMe)RKKRRQRRRG-NH_2
CTAT[L-^{Me}Ar-OMe]: CK(L-^{Me}Ar-OMe)RKKRRQRRRG-NH_2
dTAT[Tb·L-^{Me}Ar-OMe]: **(**CK(Tb·L-^{Me}Ar-OMe)RKKRRQRRRG-NH_2)$_2$ (disulfide bridge)

mTAT[L-^{Me}Ar-NHAc]: Ac-K(L-^{Me}Ar-NHAc)RKKRRQRRRG-NH_2
mTAT[Eu·L-^{Me}Ar-NHAc]: Ac-K(Eu·L-^{Me}Ar-NHAc)RKKRRQRRRG-NH_2
mTAT[Tb·L-^{Me}Ar-NHAc]: Ac-K(Tb·L-^{Me}Ar-NHAc)RKKRRQRRRG-NH_2
mTAT[Gd·L-^{Me}Ar-NHAc]: Ac-K(Gd·L-^{Me}Ar-NHAc)RKKRRQRRRG-NH_2
CTAT[L-^{Me}Ar-NHAc]: CK(L-^{Me}Ar-NHAc)RKKRRQRRRG-NH_2
dTAT[Tb·L-^{Me}Ar-NHAc]: **(**CK(Tb·L-^{Me}Ar-NHAc)RKKRRQRRRG-NH_2)$_2$ (disulfide bridge)

mTAT[L-Ar-NHAc]: Ac-K(L-^{Me}Ar-NHAc)RKKRRQRRRG-NH_2
mTAT[Eu·L-Ar-NHAc]: Ac-K(Eu·L-^{Me}Ar-NHAc)RKKRRQRRRG-NH_2
mTAT[Tb·L-Ar-NHAc]: Ac-K(Tb·L-^{Me}Ar-NHAc)RKKRRQRRRG-NH_2
mTAT[Gd·L-Ar-NHAc]: Ac-K(Gd·L-^{Me}Ar-NHAc)RKKRRQRRRG-NH_2
CTAT[L-Ar-NHAc]: CK(L-^{Me}Ar-NHAc)RKKRRQRRRG-NH_2
dTAT[Tb·L-Ar-NHAc]: **(**CK(Tb·L-^{Me}Ar-NHAc)RKKRRQRRRG-NH_2)$_2$ (disulfide bridge)

CTAT[L-^{Me}Ar-NHβAla(+)]: CK(L-^{Me}Ar-NHβAla(+))RKKRRQRRRG-NH_2
dTAT[Tb·L-^{Me}Ar-NHβAla(+)]: **(**CK(Tb·L-^{Me}Ar-NHβAla(+))RKKRRQRRRG-NH_2)$_2$ (disulfide bridge)

CTAT[L-^{Me}Ar-NHSuc(–)]: CK(L-^{Me}Ar-NHSuc(–))RKKRRQRRRG-NH_2
dTAT[Tb·L-^{Me}Ar-NHSuc(–)]: **(**CK(Tb·L-^{Me}Ar-NHSuc(–))RKKRRQRRRG-NH_2)$_2$ (disulfide bridge)

Peptide synthesis

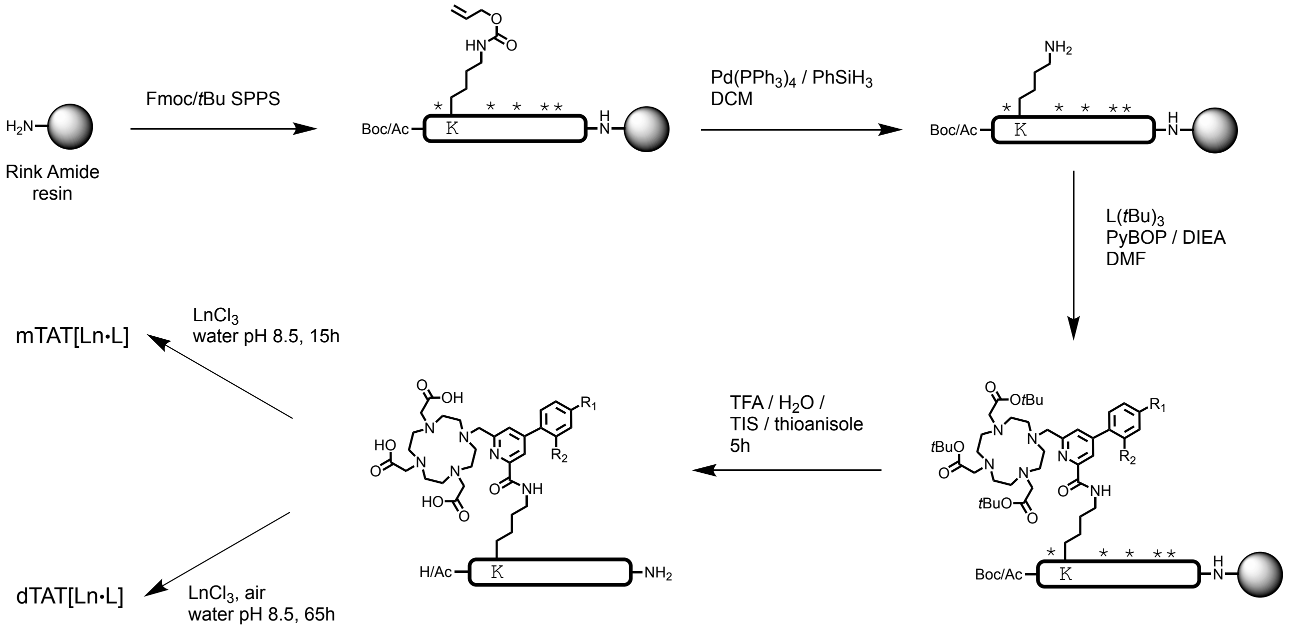

Fig. S2 Synthetic pathways for conjugates **mTAT[Ln·L]** and **dTAT[Ln·L]**. * denotes side chain protecting standard groups for Fmoc/tBu SPPS.

Peptide elongation: Peptide elongation was performed using standard SPPS procedure using Fmoc/*t*Bu chemistry on an automated peptide synthesizer (CEM Liberty1 Microwave Peptide Synthesizer) under microwave irradiation following protocols established by CEM[6] with double coupling. Fmoc removal was performed using 20 % piperidine in DMF. Couplings were performed using 4-fold molar excess of Fmoc-L-amino acid (0.2 M in DMF), 3.6-fold molar excess of HCTU (0.45 M in DMF) and 8-fold molar excess of DIEA (2 M in NMP).

AcK-TAT-RA resin: The peptide was synthesized on Rink-PEG-PS resin (NovaPEG Rink Amide, 0.25 mmol, 0.4 mmol/g). Automated peptide elongation was performed as described above using Fmoc-Lys(Alloc)-OH to introduce the N-terminal lysine. After the final Fmoc removal, the N-terminus was acetylated using Ac_2O/pyridine/DMF (1:2:7 v/v/v, 10 mL, 5 min). Removal of the Alloc protecting group was performed by adding a solution of *tetrakis*(triphenylphosphine)palladium(0) (0.073 mmol, 0.34 eq., 85 mg) and phenylsilane (12.5 mmol, 50 eq., 1.5 mL) in DCM (10 mL) for 1 h in the dark (twice).[7] The resin was then washed successively with DCM (2×2 min), DMF (2×2 min), 1 % H_2O in DMF (2×2 min), DMF (2×2 min), 1 % DIEA in DMF (2×2 min), DMF (2×2 min), sodium diethyldithiocarbamate in DMF (0.12 M, 2×5 min), DMF (2×2 min). The resin was dried and stored at -20 °C.

CK-TAT-RA resin: The peptide was synthesized on Rink-PEG-PS resin (NovaPEG Rink Amide, 0.25 mmol, 0.4 mmol/g). Automated peptide elongation was performed as described above using Fmoc-Lys(Alloc)-OH to introduce the last lysine and Boc-Cys(Trt)-OH to introduce the N-terminal cysteine. Removal of the Alloc protecting group was performed as described above for **AcK-TAT-RA resin**.

General procedure for mTAT[L] and CTAT[L]: The synthesis was performed on the 10 µmol scale. **L(*t*Bu)₃** (15 µmol, 1.5 eq.) was coupled to the peptide with PyBOP (50 µmol, 5 eq.) and DIEA (50 µL, 30 eq) in DMF (2 mL). Removal of acid-labile side chain protecting groups and resin cleavage were performed using TFA/H_2O/TIS/thioanisole (91:3:3:3 v/v/v/v, 2 mL) for 4 h. The solvent was evaporated under reduced pressure and cold Et_2O was added to precipitate the peptide. The suspension was centrifuged, the solid was washed twice with Et_2O and purified by HPLC. After freeze-drying, the conjugate was obtained as a white powder (*ca.* 5-10 mg).

mTAT[L-^{Me}Ar-OMe]: HPLC (anal.): t_R = 6.4 min (method B); LRMS (ESI+): average *m/z* = 1076.2 (2+), 717.5 (3+),

538.7 (4+), 431.2 (5+), 359.5 (6+) / calculated av. *m/z* = 1075.78 $[M+2H]^{2+}$, 717.51 $[M+3H]^{3+}$, 538.39 $[M+4H]^{4+}$, 430.91 $[M+5H]^{5+}$, 359.26 $[M+6H]^{6+}$ for M = $C_{92}H_{161}N_{39}O_{21}$); deconvoluted mass found = 2150.4 / expected mass = 2149.52 (average isotopic composition).

CTAT[L-^{Me}Ar-OMe]: HPLC (anal.): t_R = 6.3 min (method B); LRMS (ESI+): average *m/z* = 1106.3 (2+), 738.0 (3+), 553.9 (4+), 444.3 (5+), 369.7 (6+) / calculated av. *m/z* = 1106.32 $[M+2H]^{2+}$, 737.88 $[M+3H]^{3+}$, 553.66 $[M+4H]^{4+}$, 443.13 $[M+5H]^{5+}$, 369.44 $[M+6H]^{6+}$ for M = $C_{93}H_{164}N_{40}O_{21}S$); deconvoluted mass found = 2211.6 / expected mass = 2210.62 (average isotopic composition).

mTAT[L-^{Me}Ar-NHAc]: HPLC (anal.): t_R = 5.8 min (method B); LRMS (ESI+): average *m/z* = 726.6 (3+), 545.3 (4+), 436.5 (5+), 363.9 (6+), 312.1 (7+) / calculated av. *m/z* = 726.52 $[M+3H]^{3+}$, 545.14 $[M+4H]^{4+}$, 436.32 $[M+5H]^{5+}$, 363.76 $[M+6H]^{6+}$, 311.94 $[M+7H]^{7+}$ for M = $C_{93}H_{162}N_{40}O_{21}$); deconvoluted mass found = 2277.2 / expected mass = 2176.54 (average isotopic composition).

CTAT[L-^{Me}Ar-NHAc]: HPLC (anal.): t_R = 5.8 min (method B); LRMS (ESI+): average *m/z* = 746.9 (3+), 560.6 (4+), 448.8 (5+), 374.1 (6+) / calculated av. *m/z* = 746.89 $[M+3H]^{3+}$, 560.42 $[M+4H]^{4+}$, 448.54 $[M+5H]^{5+}$, 373.95 $[M+6H]^{6+}$ for M = $C_{94}H_{165}N_{41}O_{21}S$); deconvoluted mass found = 2238.9 / expected mass = 2237.65 (average isotopic composition).

mTAT[L-Ar-NHAc]: HPLC (anal.): t_R = 5.7 min (method B); LRMS (ESI+): average *m/z* = 722.0 (3+), 541.8 (4+), 433.8 (5+), 361.7 (6+) / calculated av. *m/z* = 721.85 $[M+3H]^{3+}$, 541.64 $[M+4H]^{4+}$, 433.51 $[M+5H]^{5+}$, 361.43 $[M+6H]^{6+}$ for M = $C_{92}H_{160}N_{40}O_{21}$); deconvoluted mass found = 2163.6 / expected mass = 2162.52 (average isotopic composition).

CTAT[L-Ar-NHAc]: HPLC (anal.): t_R = 5.6 min (method B); LRMS (ESI+): average *m/z* = 742.0 (3+), 556.8 (4+), 445.7 (5+) / calculated av. *m/z* = 742.22 $[M+3H]^{3+}$, 556.91 $[M+4H]^{4+}$, 445.73 $[M+5H]^{5+}$, 371.61 $[M+6H]^{6+}$ for M = $C_{93}H_{163}N_{41}O_{21}S$); deconvoluted mass found = 2223.2 / expected mass = 2223.62 (average isotopic composition).

CTAT[L-^{Me}Ar-NHβAla(+)]: HPLC (anal.): t_R = 5.3 min (method B); LRMS (ESI+): average *m/z* = 756.5 (4+), 567.8 (4+), 454.4 (5+), 378.9 (6+), 324.9 (7+) / calculated av. *m/z* = 756.57 $[M+3H]^{3+}$, 567.67 $[M+4H]^{4+}$, 454.34 $[M+5H]^{5+}$, 378.79 $[M+6H]^{6+}$, 324.82 $[M+7H]^{7+}$ for M = $C_{95}H_{168}N_{42}O_{21}S$); deconvoluted mass found = 2266.9 / expected mass = 2266.68 (average isotopic composition).

CTAT[L-^{Me}Ar-NHSuc(–)]: HPLC (anal.): t_R = 5.6 min (method B); LRMS (ESI+): average *m/z* = 766.2 (3+), 575.0 (4+), 460.3 (5+), 383.8 (6+) / calculated av. *m/z* = 766.23 $[M+3H]^{3+}$, 574.93 $[M+4H]^{4+}$, 460.14 $[M+5H]^{5+}$, 383.62 $[M+6H]^{6+}$ for M = $C_{96}H_{167}N_{41}O_{23}S$); deconvoluted mass found = 2296.7 / expected mass = 2295.68 (average isotopic composition).

General procedure for mTAT[LnL] complexes: Compound **mTAT[L]** (*ca.* 3 mg) and $LnCl_3\cdot(H_2O)_6$ (10 eq.) were dissolved in H_2O (0.6 mL) and the pH was adjusted to 7.5 using NaOH. The solution was stirred overnight at room temperature. The pH was decreased to *ca.* 3 using HCl before HPLC purification. After freeze-drying, **mTAT[LnL]** was obtained as a white powder (90 %).

mTAT[Eu·L-^{Me}Ar-OMe]: HPLC (anal.): t_R = 6.4 min (method B); LRMS (ESI+): average *m/z* = 767.6 (3+), 575.9 (4+), 460.9 (5+), 384.3 (6+) / calculated av. *m/z* = 767.16 $[M+3H]^{3+}$, 575.62 $[M+4H]^{4+}$, 460.70 $[M+5H]^{5+}$, 384.08 $[M+6H]^{6+}$ for M = $C_{92}H_{158}N_{39}O_{21}Eu$); deconvoluted mass found = 2299.2 / expected mass = 2298.45 (average isotopic composition).

mTAT[Tb·L-^{Me}Ar-OMe]: HPLC (anal.): t_R = 6.3 min (method B); LRMS (ESI+): average *m/z* = 769.7 (3+), 577.8 (4+), 462.3 (5+), 385.5 (6+) / calculated av. *m/z* = 769.48 $[M+3H]^{3+}$, 577.36 $[M+4H]^{4+}$, 462.09 $[M+5H]^{5+}$, 385.24 $[M+6H]^{6+}$ for M = $C_{92}H_{158}N_{39}O_{21}Tb$); deconvoluted mass found = 2306.7 / expected mass = 2305.42 (average isotopic composition).

mTAT[Gd·Eu·L-^{Me}Ar-OMe]: HPLC (anal.): t_R = 6.3 min (method B); LRMS (ESI+): average *m/z* = 769.0 (3+), 577.2 (4+), 462.0 (5+), 385.3 (6+) / calculated av. *m/z* = 768.92 $[M+3H]^{3+}$, 576.95 $[M+4H]^{4+}$, 461.76 $[M+5H]^{5+}$, 384.96 $[M+6H]^{6+}$ for M = $C_{92}H_{158}N_{39}O_{21}Gd$); deconvoluted mass found = 2305.1 / expected mass = 2303.74 (average isotopic composition).

mTAT[Eu·L-^{Me}Ar-NHAc]: HPLC (anal.): t_R = 5.8 min (method B); LRMS (ESI+): average *m/z* = 776.5 (3+), 582.6 (4+), 466.3 (5+), 388.8 (6+) / calculated av. *m/z* = 776.17 $[M+3H]^{3+}$, 582.38 $[M+4H]^{4+}$, 466.10 $[M+5H]^{5+}$, 388.59 $[M+6H]^{6+}$ for M = $C_{93}H_{159}N_{40}O_{21}Eu$); deconvoluted mass found = 2327.1 / expected mass = 2325.48 (average isotopic

composition).

mTAT[Tb·L-^{Me}Ar-NHAc]: HPLC (anal.): t_R = 5.8 min (method B); LRMS (ESI+): average *m/z* = 778.6 (3+), 584.3 (4+), 467.7 (5+), 388.9 (6+) / calculated av. *m/z* = 778.49 $[M+3H]^{3+}$, 584.12 $[M+4H]^{4+}$, 467.50 $[M+5H]^{5+}$, 389.75 $[M+6H]^{6+}$ for M = $C_{93}H_{159}N_{40}O_{21}Tb$); deconvoluted mass found = 2333.3 / expected mass = 2332.44 (average isotopic composition).

mTAT[Gd·L-^{Me}Ar-NHAc]: HPLC (anal.): t_R = 5.7 min (method B); LRMS (ESI+): average *m/z* = 777.9 (3+), 583.8 (4+), 467.3 (5+), 389.7 (6+) / calculated av. *m/z* = 777.93 $[M+3H]^{3+}$, 583.70 $[M+4H]^{4+}$, 467.16 $[M+5H]^{5+}$, 389.47 $[M+6H]^{6+}$ for M = $C_{93}H_{159}N_{40}O_{21}Gd$); deconvoluted mass found = 2321.2 / expected mass = 2330.77 (average isotopic composition).

mTAT[Eu·L-Ar-NHAc]: HPLC (anal.): t_R = 5.6 min (method B); LRMS (ESI+): average *m/z* = 771.8 (3+), 579.0 (4+), 463.5 (5+), 386.4 (6+) / calculated av. *m/z* = 771.49 $[M+3H]^{3+}$, 578.87 $[M+4H]^{4+}$, 463.30 $[M+5H]^{5+}$, 386.25 $[M+6H]^{6+}$ for M = $C_{92}H_{157}N_{40}O_{21}Eu$); deconvoluted mass found = 2311.5 / expected mass = 2311.46 (average isotopic composition).

mTAT[Tb·L-Ar-NHAc]: HPLC (anal.): t_R = 5.7 min (method B); LRMS (ESI+): average *m/z* = 580.8 (4+), 464.8 (5+), 387.6 (6+) / calculated av. *m/z* = 580.61 $[M+4H]^{4+}$, 464.69 $[M+5H]^{5+}$, 387.41 $[M+6H]^{6+}$ for M = $C_{92}H_{157}N_{40}O_{21}Tb$); deconvoluted mass found = 2319.0 / expected mass = 2318.42 (average isotopic composition).

mTAT[Gd·L-Ar-NHAc]: HPLC (anal.): t_R = 5.6 min (method B); LRMS (ESI+): average *m/z* = 580.3 (4+), 464.4 (5+), 387.3 (6+) / calculated av. *m/z* = 580.19 $[M+4H]^{4+}$, 464.36 $[M+5H]^{5+}$, 387.13 $[M+6H]^{6+}$ for M = $C_{92}H_{157}N_{40}O_{21}Gd$); deconvoluted mass found = 2317.0 / expected mass = 2316.74 (average isotopic composition).

General procedure for dTAT[LnL] complexes: Compound **CTAT[L]** (*ca.* 3-5 mg) and $LnCl_3{\cdot}(H_2O)_6$ (10 eq.) were dissolved in H_2O (0.6 mL) and the pH was adjusted to 8.5 using NaOH. The solution was stirred under air at room temperature and LCMS was used to monitor completeness of the metalation and of the formation of the disulfide bridge (slower). After 48-64 h on stirring, the pH was decreased to *ca.* 3 using HCl before HPLC purification. After freeze-drying, **dTAT[LnL]** was obtained as a white powder (90 %).

dTAT[Tb·L-^{Me}Ar-OMe]: HPLC (anal.): t_R = 6.6 min (method B); LRMS (ESI+): average *m/z* = 793.8 (6+), 677.2 (7+), 592.7 (8+), 526.9 (9+), 474.4 (10+), 431.3 (11+), 395.5 (12+) / calculated av. *m/z* = 947.21 [M+5H]5+, 789.51 $[M+6H]^{6+}$, 676.87 $[M+7H]^{7+}$, 592.39 $[M+8H]^{8+}$, 526.68 $[M+9H]^{9+}$, 474.11 $[M+10H]^{10+}$, 431.10 $[M+11H]^{11+}$, 395.26 $[M+12H]^{12+}$ for M = $C_{186}H_{320}N_{80}O_{42}S_2Tb_2$); deconvoluted mass found = 4733.4 / expected mass = 4731.04 (average isotopic composition).

dTAT[Tb·L-^{Me}Ar-NHAc]: HPLC (anal.): t_R = 6.1 min (method B); LRMS (ESI+): average *m/z* = 798.8 (6+), 684.8 (7+), 599.4 (8+), 532.8 (9+), 479.8 (10+), 436.3 (11+), 400.0 (12+) / calculated av. *m/z* = 798.52 $[M+6H]^{6+}$, 684.59 $[M+7H]^{7+}$, 599.14 $[M+8H]^{8+}$, 532.68 $[M+9H]^{9+}$, 479.52 $[M+10H]^{10+}$, 436.02 $[M+11H]^{11+}$, 399.76 $[M+12H]^{12+}$ for M = $C_{188}H_{322}N_{82}O_{42}S_2Tb_2$); deconvoluted mass found = 4786.6 / expected mass = 4786.09 (average isotopic composition).

dTAT[Eu·L-Ar-NHAc]: HPLC (anal.): t_R = 5.9 min (method B); LRMS (ESI+): average *m/z* = 949.8 (5+), 791.8 (6+), 678.8 (7+), 594.3 (8+), 528.3 (9+), 475.3 (10+), 432.3 (11+), 396.3 (12+) / calculated av. *m/z* = 949.63 $[M+5H]^{5+}$, 791.53 $[M+6H]^{6+}$, 678.59 $[M+7H]^{7+}$, 593.90 $[M+8H]^{8+}$, 528.02 $[M+9H]^{9+}$, 475.32 $[M+10H]^{10+}$, 432.20 $[M+11H]^{11+}$, 396.27 $[M+12H]^{12+}$ for M = $C_{186}H_{318}N_{82}O_{42}S_2Eu_2$); deconvoluted mass found = 4744.6 / expected mass = 4743.11 (average isotopic composition).

dTAT[Tb·L-^{Me}Ar-NHβAla(+)]: HPLC (anal.): t_R = 5.6 min (method B); LRMS (ESI+): average *m/z* = 693.0 (7+), 606.6 (8+), 539.3 (9+), 485.5 (10+), 441.5 (11+), 404.8 (12+) / calculated av. *m/z* = 692.48 $[M+7H]^{7+}$, 606.40 $[M+8H]^{8+}$, 539.14 $[M+9H]^{9+}$, 485.32 $[M+10H]^{10+}$, 441.29 $[M+11H]^{11+}$, 404.60 $[M+12H]^{12+}$ for M = $C_{190}H_{328}N_{84}O_{42}S_2Tb_2$); deconvoluted mass found = 4844.6 / expected mass = 4843.15 (average isotopic composition).

dTAT[Tb·L-^{Me}Ar-NHSuc(–)]: HPLC (anal.): t_R = 6.0 min (method B); LRMS (ESI+): average *m/z* = 818.0 (6+), 701.3 (7+), 613.9 (8+), 545.8 (9+), 491.3 (10+) / calculated av. *m/z* = 817.86 $[M+6H]^{6+}$, 701.17 $[M+7H]^{7+}$, 613.65 $[M+8H]^{8+}$, 545.58 $[M+9H]^{9+}$, 491.12 $[M+10H]^{10+}$ for M = $C_{192}H_{326}N_{82}O_{46}S_2Tb_2$); deconvoluted mass found = 4902.3 / expected mass = 4901.13 (average isotopic composition).

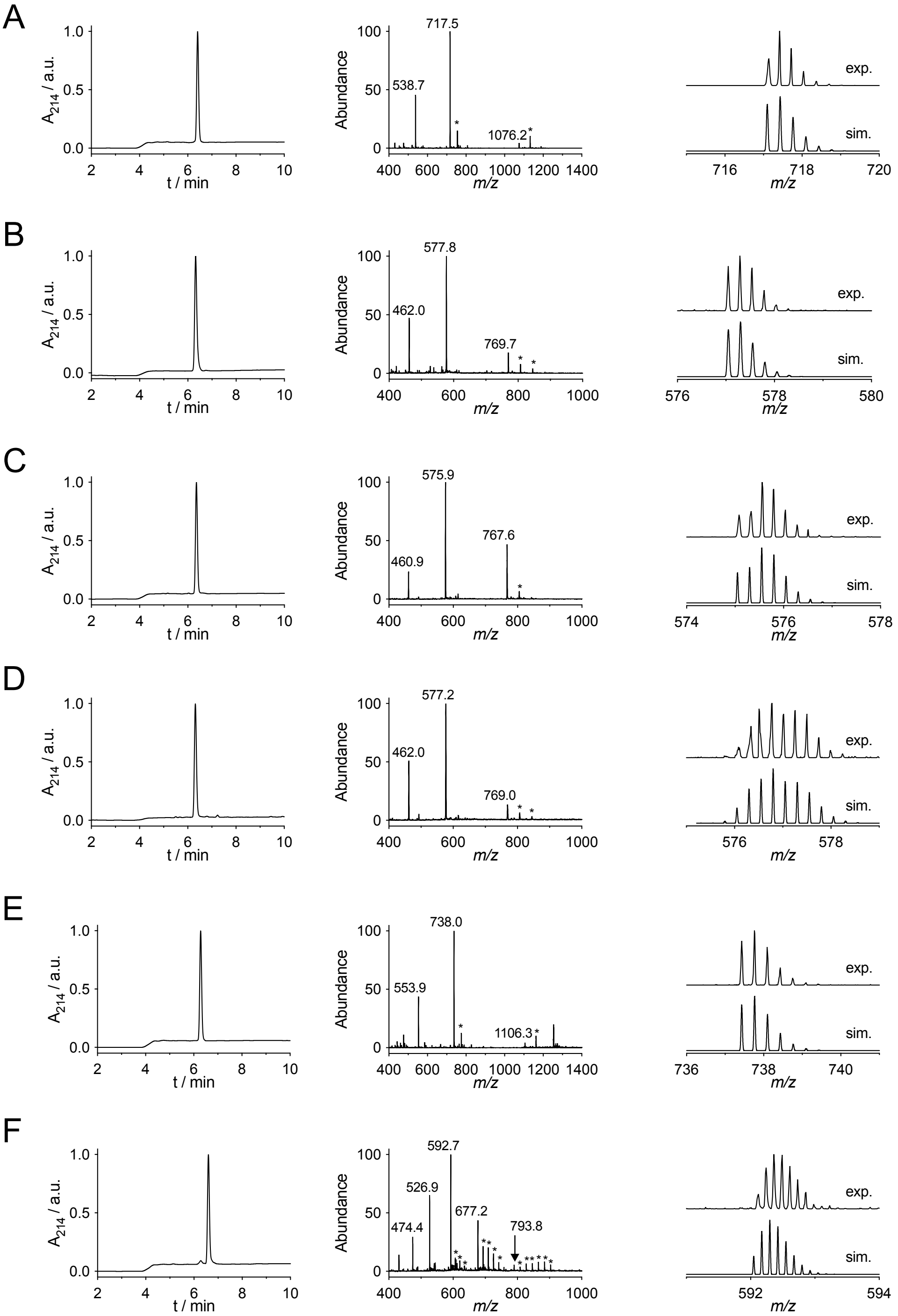

Fig. S3 Typical examples of HPLC chromatograms (*left*) and LRMS (ESI+) spectra (*middle*: full MS spectrum; *right*: experimental and simulated isotopic pattern) obtained for (A) **mTAT[L-^{Me}Ar-OMe]**, (B) **mTAT[Tb·L-^{Me}Ar-OMe]**, (C) **mTAT[Eu·L-^{Me}Ar-OMe]**, (D) **mTAT[Gd·L-^{Me}Ar-OMe]**, (E) **CTAT[L-^{Me}Ar-OMe]** and (F) **dTAT[Tb·L-^{Me}Ar-OMe]**. In full MS spectra, * indicates a TFA adduct.

1P spectroscopy

Determination of molar extinction coefficients: A solution of mTAT[L] or CTAT[L] peptide in HEPES buffer (10 mM, pH 7.5) was titrated by a solution of $TbCl_3$ or $EuCl_3$ of known concentration with absorption monitoring, as previously described.[8] The concentration of the peptide was determined from the endpoint of the titration, allowing determination of extinction coefficient using Beer Lambert law. The titration of mTAT[L-^{Me}Ar-OMe] by Tb^{3+} is given in Fig. S4 as typical example.

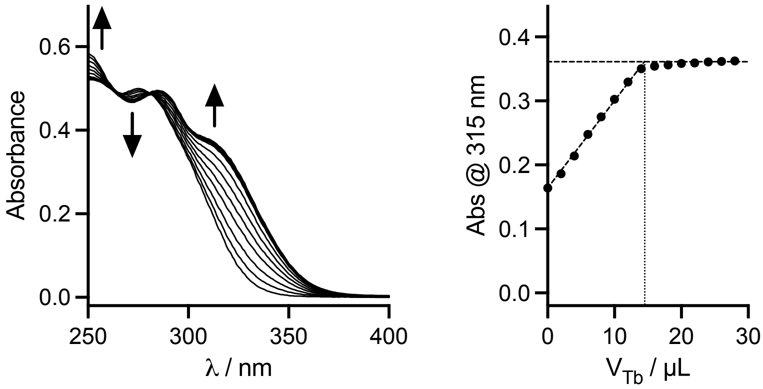

Fig. S4 Absorbance titration of mTAT[L-^{Me}Ar-OMe] in HEPES buffer (10 mM, pH 7.5, 1 mL) by $TbCl_3$ in H_2O (2.35 mM). Pathlength is 1.0 cm.

Absorption, excitation and emission spectra:

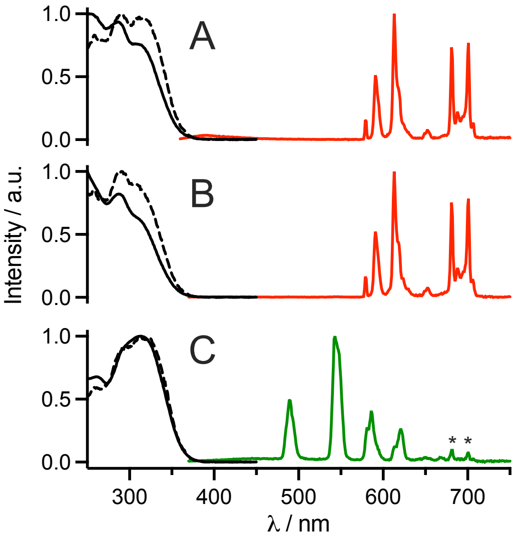

Fig. S5 Normalized absorption (black solid line), excitation (black dashed line; λ_{em} = 545 nm (A, B) or 615 nm (C)) and emission (colored solid line; λ_{ex} = 315 mn) spectra of (A) mTAT[Eu·L-^{Me}Ar-OMe], (B) mTAT[Eu·L-^{Me}Ar-NHAc] and (C) mTAT[Tb·L-Ar-NHAc] in PBS. In (C), * denotes Eu^{3+} contamination.

Luminescence decay: Ln^{3+} luminescence decays were measured for each compound in aerated and de-oxygenated PBS, prepared in H_2O, in aerated PBS prepared in D_2O and lifetimes, τ_{Ln}, were determined by mono-exponential fit (or bi-exponential fit when required). They are indicated in Table S1 as well as hydration number q determined using Parker's equations: $q^{Tb} = 5.0 \times (1/\tau(H_2O) - 1/\tau(D_2O) - 0.06)$ and $q^{Eu} = 1.2 \times (1/\tau(H_2O) - 1/\tau(D_2O) - 0.325))$ for Tb^{3+} and Eu^{3+},respectively, with τ in ms.[9] Examples of decay curves and their fits are given in Fig. S6.

Table S1: Luminescence lifetimes and hydration numbers *q* of mTAT[Ln·L] conjugates in PBS (λ_{ex} = 315 nm).[a]

Compound	Ln	τ_{Ln} / ms H2O, aerated	τ_{Ln} / ms H2O, de-oxygenated	τ_{Ln} / ms D2O, aerated	*q* (± 0.2)
mTAT[Ln·L-^{Me}Ar-OMe]	Tb	2.11	2.38	2.41	0.0
	Eu	1.05	1.05	1.65	0.0
mTAT[Ln·L-^{Me}Ar-NHAc]	Tb	0.83	1.02	1.03	n.d.[b]
	Eu	1.05	1.05	1.65	0.0
mTAT[Ln·L-Ar-NHAc]	Tb	0.07	0.20	0.08	n.d.[b]
	Eu	1.05	1.05	1.63	0.0

[a] Error on τ and *q* values are estimated ± 0.03 ms and ± 0.2, respectively. [b] Since back energy transfer to the triplet state contributes significantly to Tb^{3+} $^5D_4$ state decay, estimation of *q* by Parker's equation is not reliable and thus, *q* was not calculated.

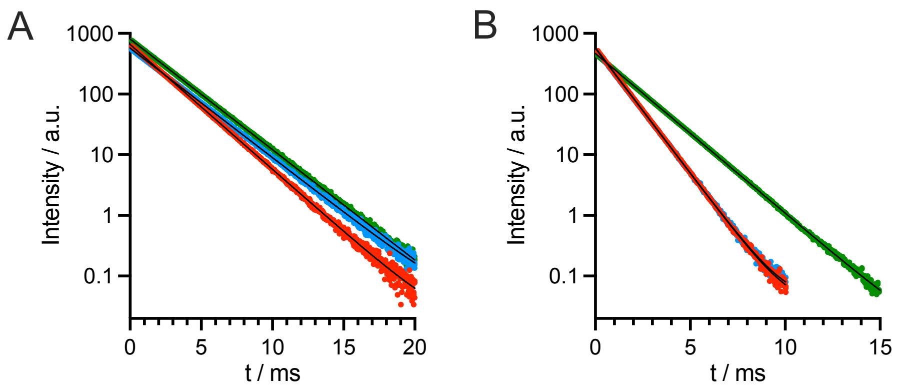

Fig. S6 Typical examples of luminescence decays and their mono-exponential fits measured for (A) mTAT[Tb·L-^{Me}Ar-OMe] and (B) mTAT[Eu·L-^{Me}Ar-OMe] in PBS (red: H_2O solution, aerated; blue: H_2O solution, de-oxygenated; green: D_2O solution, aerated). Lifetimes obtained by mono-exponential fits are given in Table S1.

Quantum yields measurements: Quantum yields were determined using a Fluorolog FL3-22 spectrophotometer by a relative method with quinine sulphate in 0.5 M H_2SO_4 as a reference compound (Φ = 0.545)[10,11] using solutions of various concentrations having absorption below 0.1 at the excitation wavelength. The excitation wavelength was the same for the sample compound (S) and the reference. To determine the quantum yields of the sample compound, the following equation was used:

$$\Phi_S = \frac{A_S}{I_S} \times \frac{I_{ref}}{A_{ref}} \times \frac{{n_S}^2}{{n_{ref}}^2} \times \Phi_{ref} \qquad (1)$$

where *A* is the absorbance at the excitation wavelength, *I* the integrated emission intensity and *n* the refractive index. Estimated experimental error for the quantum yield determination is ~10 %. Fig. S7 shows data for mTAT[Ln·L-^{Me}Ar-OMe] as typical examples.

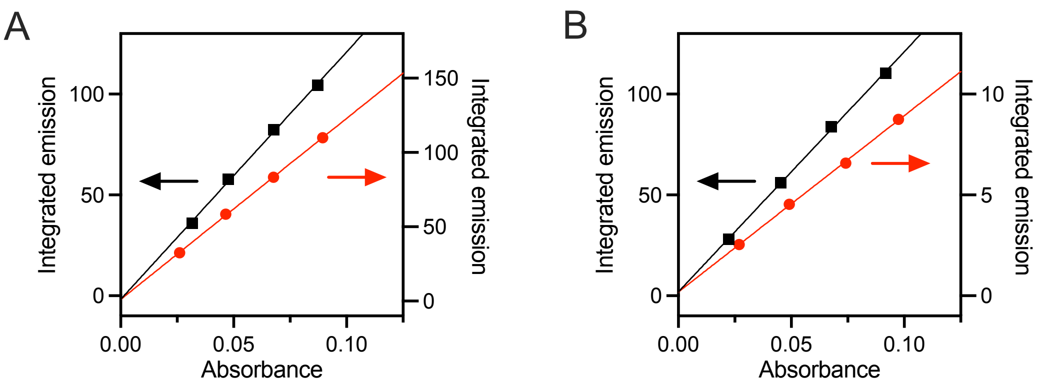

Fig. S7 Variation of integrated intensity against absorbance for (A) mTAT[Tb·L-^{Me}Ar-OMe] and (B) mTAT[Eu·L-^{Me}Ar-OMe] in PBS (red: conjugate; black: quinine sulfate). The slopes that correspond to *I*/*A* in Equation (1) were used to determine the quantum yields.

Determination of energy of S_1 and T_1 excited states: The energy of the S_1 state was determined using the cut-off of the absorption band. For the energy of T_1 excited state, a solution of the Gd-loaded peptide in PBS/glycerol 9:1 (v/v) was prepared and frozen at 77 K. A time-gated (delay = 100 µs) emission spectrum was recorded to monitor the antenna phosphorescence emission. The energy of the T_1 state was estimated using the wavelength at half-maximum on the onset of the phosphorescence spectrum.

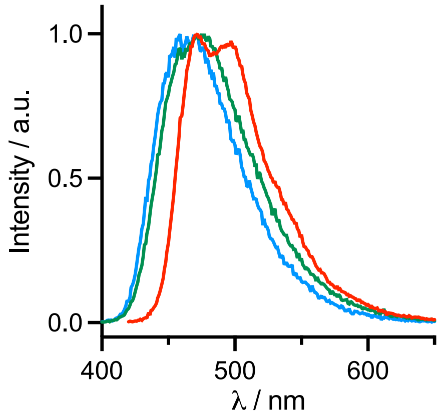

Fig. S8 Time-gated (100 µs delay) emission spectrum of mTAT[Gd·L-^{Me}Ar-OMe] (blue), mTAT[Gd·L-^{Me}Ar-NHAc] (green) and mTAT[Gd·L-Ar-NHAc] (red) in PBS/glycerol 9:1 (v/v) at 77 K (frozen solutions).

2P spectroscopy

Determination of 2P cross-sections σ_{2P}: Two-photon excitation spectra and two-photon cross-sections were obtained by two-photon excited fluorescence measurements of diluted PBS solutions of the compounds (*ca.* 10 µM, the exact concentration was determined from absorption spectrum) using a femtosecond Ti:sapphire laser (Coherent Chameleon Ultra II, 80 MHz, 140 fs) in the range 690–990 nm. The excitation beam (2.6 mm diameter) was focused with a 75 mm focal length lens to the sample. The up-converted fluorescence was collected at right-angle using a 30 mm focal length doublet lens. After filtering the scattered excitation beam by low-pass filters, the florescence was coupled to a fiber optic spectrometer (Avantes Hero). The sample was contained in a 1×1 cm quartz cell and continuously stirred with a magnetic stirrer to avoid thermal effects and photodegradation. After verifying that the emission intensity exhibited quadratic power

dependence for each sample (Fig. S9), the incident power was adjusted to 35 mW to characterize the two-photon absorption spectra. Calibration of the 2P absorption spectra was performed at each excitation wavelength by comparison with that of fluorescein (10 µM, pH 11) as reference compound.[12] 2P absorption spectra are provided in Fig. S10.

2P microscopy

General: HeLa, MRC5 and HEK293 cell lines were purchased from the ATCC and agreed by French ethic boards (CODECOH DC-2020-4226).
Cell culture: HeLa cells were grown in RPMI medium supplemented with 10% fetal bovine serum (v/v) at 37°C in a 5% CO_2 humidified atmosphere.
Peptides delivery in live cells: HeLa cells were seeded at 3×10^4 cells/well onto an 8-chamber Labtek-I coverglass system. After 24 h, cells were washed three times with PBS. Cells were incubated with dTAT [Ln·L] (10 µM) in RPMI medium (without phenol red and without serum) or PBS at 37°C for 1 h. Then, cells were washed three times with PBS before adding RPMI medium supplemented with fetal bovine serum (200 µL) for observation under microscope.
Confocal microscopy: Confocal 2P experiments were performed by using an LSM-DuoScan-Confocor3 NLO microscope (Carl Zeiss) composed of a LSM710 confocal module and an inverted motorized stand (AxioObserver) equipped with an on-stage cell incubator. Excitation was provided by a Ti:Sapphire femtosecond laser (Chameleon, Ultra II, Coherent) featuring chirp precompensation. The C-apochromat 40×/1.2 water-immersion objective was used throughout experiments. The pinhole was open during 2P acquisition in descanned detection mode. The spectral PMT detector (Quasar) or avalanche photodiodes were used to register the emission signal in each pixel of the confocal image. Temporal Sampling Lifetime Imaging Microscopy (TSLIM) was used to record luminescence lifetime decay in cells (single pulsed excitation with the 7.56 µs temporal resolution).[13]
Image analysis: Fiji/ImageJ was used to analyze images and particularly for linear unmixing to deconvolute autofluorescence and Ln^{3+} emission maps. They were performed using the Stowers ImageJ plugins developed by Jay Unruh at the Stowers Institute for Medical Research. Pure 2P-excited autofluorescence and Ln^{3+} emission spectra used for linear unmixing were acquired with the LSM710 microscope from control HeLa cells (Fig. S9) or droplets of mTAT[Ln·L] conjugates (100 µM in PBS). They are displayed in Fig. S10.

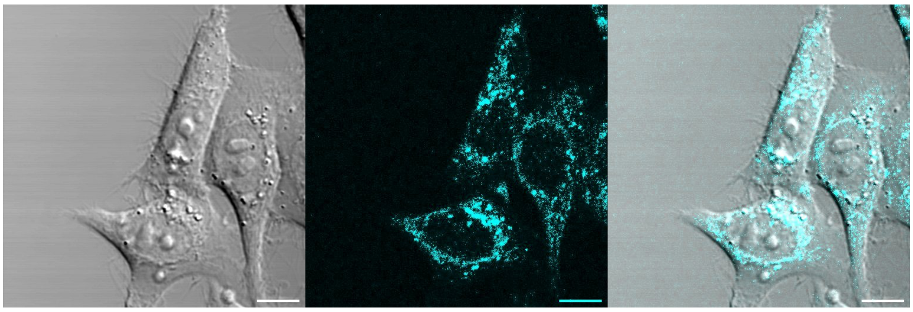

Fig. S9 2PM imaging (λ_{ex} = 720 nm) of control living HeLa cells (not incubated with dTAT[Ln·L]). Left panel shows the differential interference contrast (DIC) image, middle panel shows the luminescence image, and right panel shows the merge. Scale bar corresponds to 10 µm.

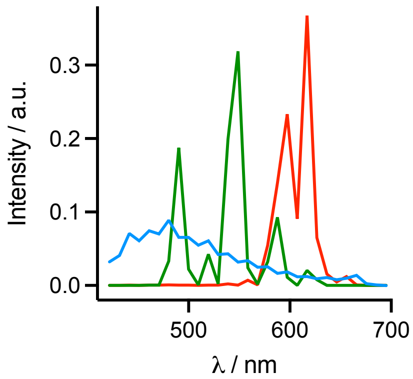

Fig. S10 2P-excited emission spectra of HeLa cell autofluorescence (blue), mTAT[Tb·L-^{Me}Ar-NHAc] (green) and mTAT[Eu·L-Ar-NHAc] (red) recorded with the LSM710 microscope (λ_{ex} = 720 nm).

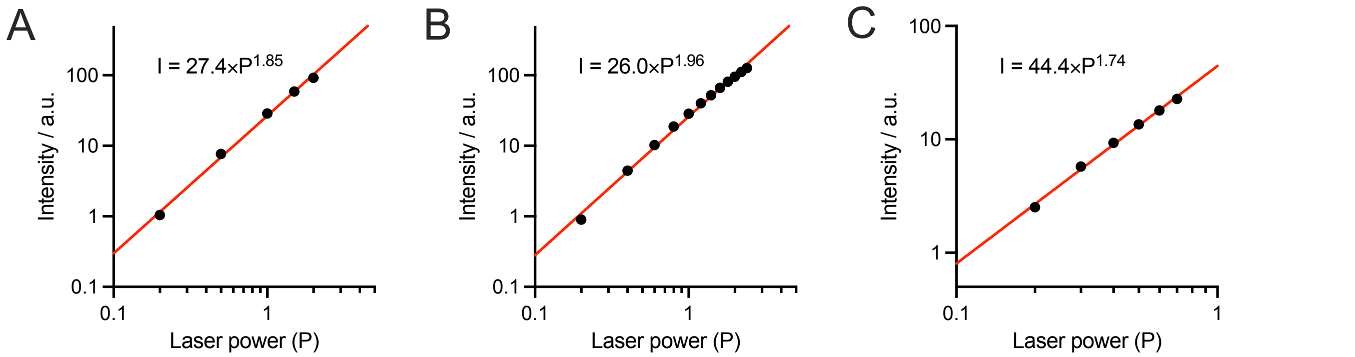

Fig. S11 Quadratic power dependence of the intensity of luminescence emission arising from HeLa cells incubated with (A) **dTAT[Tb·L-^{Me}Ar-OMe]**, (B) **dTAT[Tb·L-^{Me}Ar-NHAc]** and (C) **dTAT[Eu·L-Ar-NHAc]** (10 μM), detected with the LSM710 microscope PMT (λ_{ex} = 720 nm for A and C, 700 nm for B).

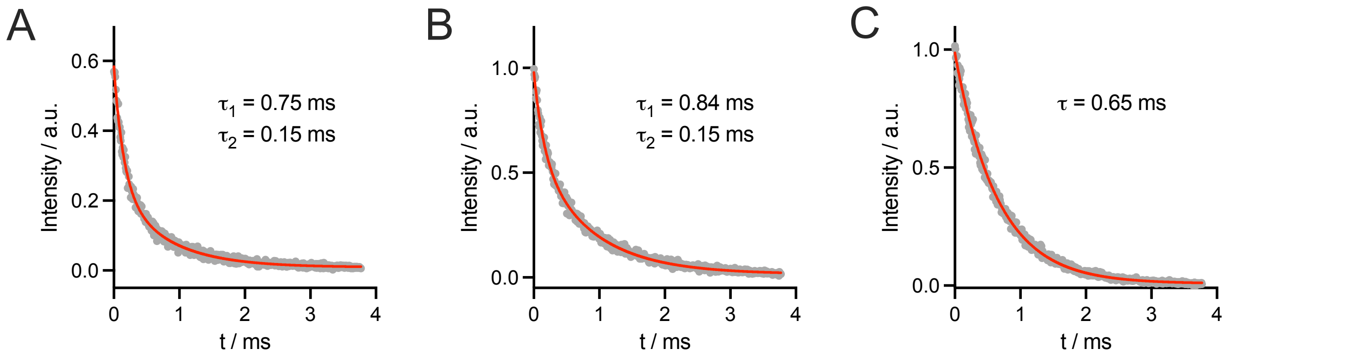

Fig. S12 Luminescence decay of (A) dTAT[Tb·L-^{Me}Ar-OMe], (B) dTAT[Tb·L-^{Me}Ar-NHAc] and (C) dTAT[Eu·L-Ar-NHAc] in HeLa cells (1 h incubation at 10 μM), recorded by TSLIM[13] with the LSM710 microscope (APD; λ_{ex} = 720 nm for A and C, 700 nm for B). Experimental data were fitted to bi-exponential (A and B) or mono-exponential (C) decays yielding τ values indicated on the graphs and in the text.

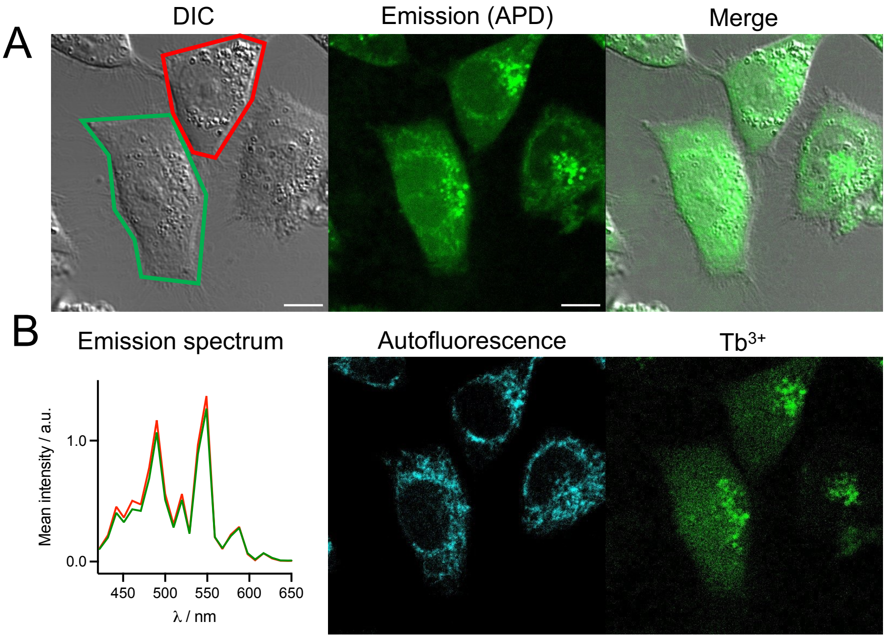

Fig. S13 2PM imaging (λ_{ex} = 720 nm) of living HeLa cells incubated 1 h with dTAT[Tb·L-^{Me}Ar-NHAc] (10 µM) in RPMI medium. (A) *Left panel:* DIC image; *Middle panel*: luminescence (detected with APD) image; *Right panel:* merge. Scale bars correspond to 10 µm. (B) *Left panel:* 2P-excited emission spectra (detection with PMT) of cells surrounded in red and green in panel A; *Middle and right panels:* autofluorescence and Tb^{3+} emission maps obtained by linear unmixing of 2P-excited spectral images recorded with the PMT.

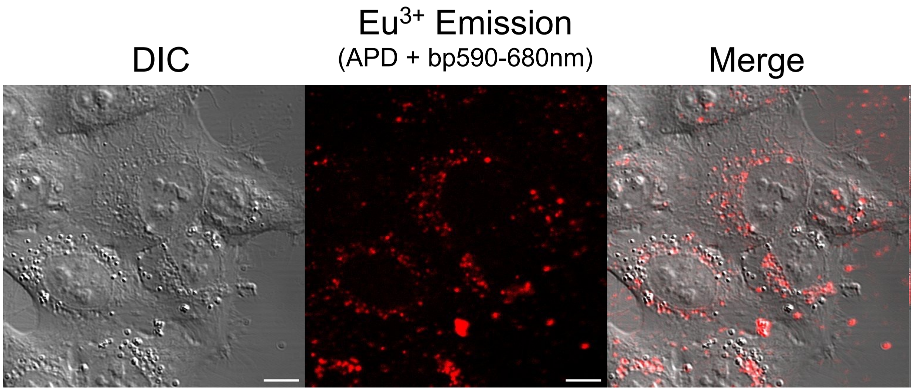

Fig. S14 2PM imaging (λ_{ex} = 720 nm) of living HeLa cells incubated 1 h with dTAT[Eu·L-Ar-NHAc] (2.5 µM) in RPMI medium. *Left panel:* DIC image; *Bottom left*: *Middle panel*: Eu^{3+} luminescence (detected with APD and a 590-680 nm band-pass filter) image; *Right panel:* merge. Scale bars correspond to 10 µm.

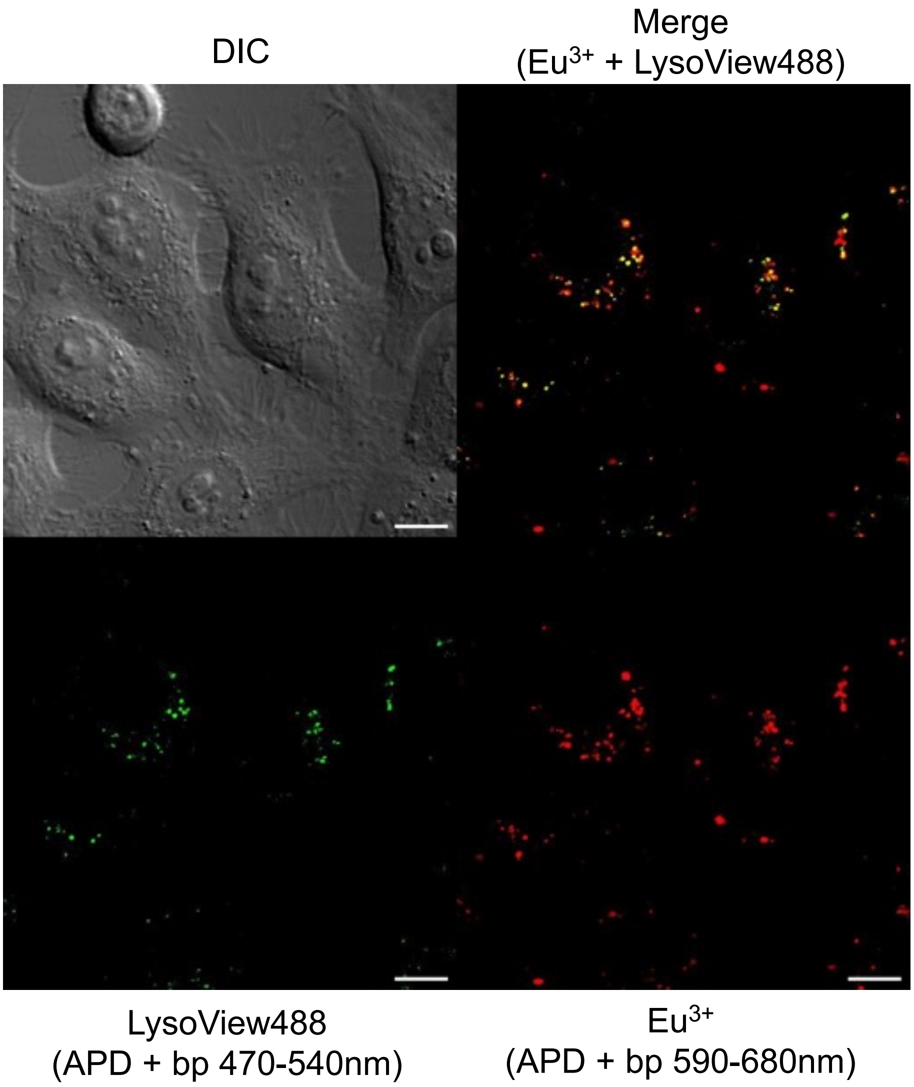

Fig. S15 2PM imaging (λ_{ex} = 720 nm) of living HeLa cells incubated 1 h with dTAT[Eu·L-Ar-NHAc] (2.5 µM) in RPMI medium and counter-stained with Lysoview488. *Top left panel:* DIC image; *Bottom left panel*: green channel / LysoView488 emission (detected with APD and a 470-540 nm band-pass filter); *Bottom right panel:* red channel / Eu3+ luminescence (detected with APD and a 590-680 nm band-pass filter); merge of LysoView488 and Eu^{3+} emission. Scale bars correspond to 10 µm. In the green channel (Lysoview488), signal intensity was scaled so that autofluorescence is not shown.

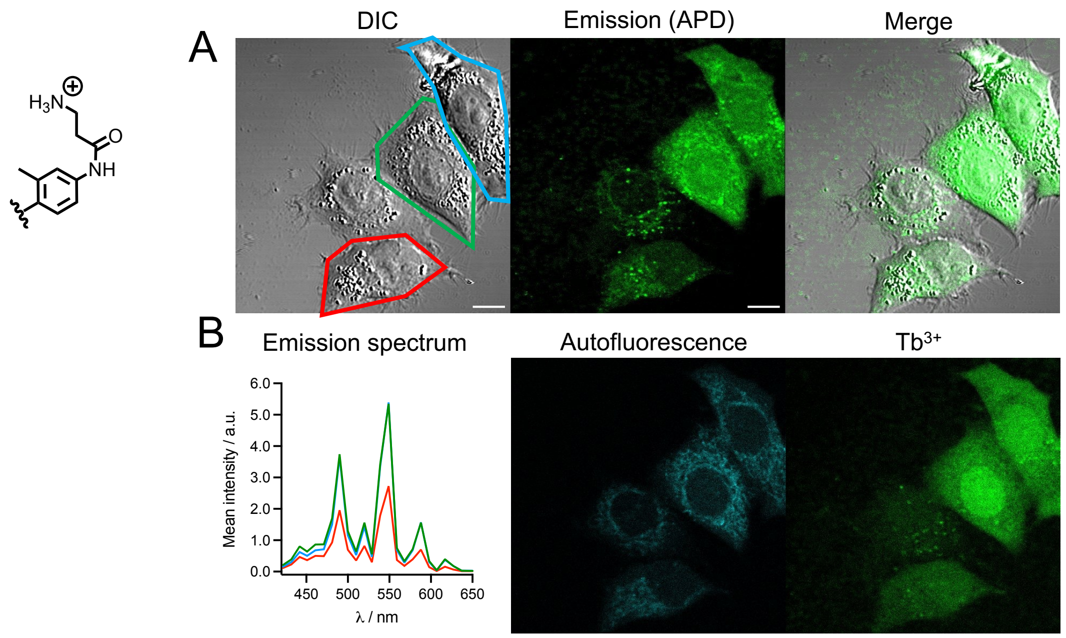

Fig. S16. 2PM imaging (λ_{ex} = 720 nm) of living HeLa cells incubated 1 h with dTAT[Tb·L-^{Me}Ar-NHβAla(+)] (10 µM) in RPMI medium. (A) *Left panel:* DIC image; *Middle panel*: luminescence (detected with APD) image; *Right panel:* merge. Scale bars correspond to 10 µm. (B) *Left panel:* 2P-excited emission spectra (detection with PMT) of cells surrounded in red, green and blue in panel A; *Middle and right panels:* autofluorescence and Tb^{3+} emission maps obtained by linear unmixing of 2P-excited spectral images recorded with the PMT.

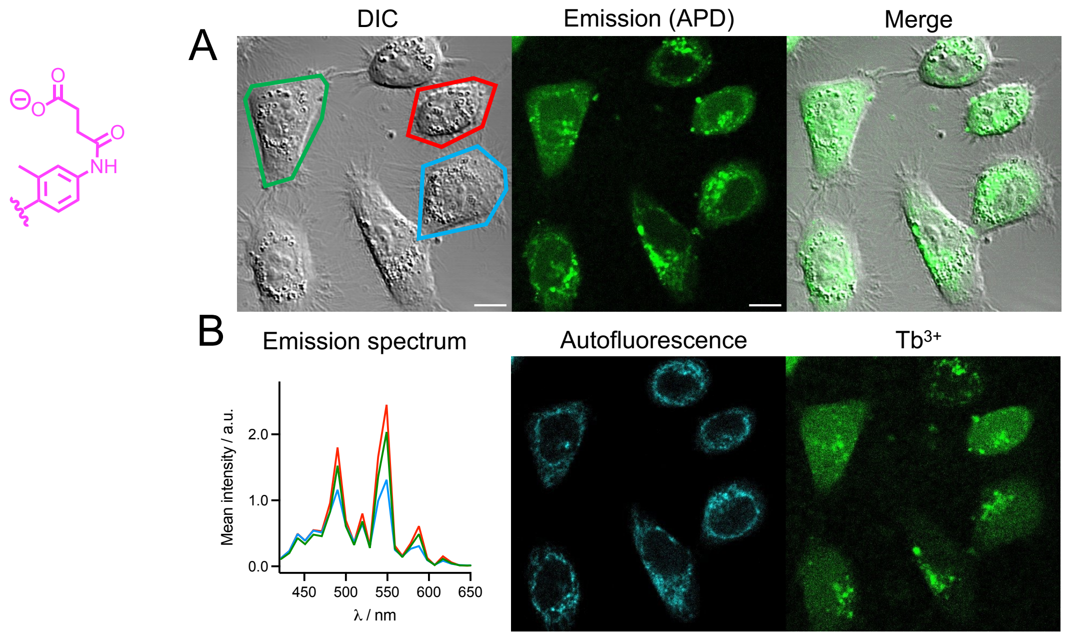

Fig. S17 2PM imaging (λ_{ex} = 720 nm) of living HeLa cells incubated 1 h with dTAT[Tb·L-^{Me}Ar-NHSuc(–)] (10 µM) in RPMI medium. (A) *Left panel:* DIC image; *Middle panel*: luminescence (detected with APD) image; *Right panel:* merge. Scale bars correspond to 10 µm. (B) *Left panel:* 2P-excited emission spectra (detection with PMT) of cells outlined in red, green and blue in panel A; *Middle and right panels:* autofluorescence and Tb^{3+} emission maps obtained by linear unmixing of 2P-excited spectral images recorded with the PMT.

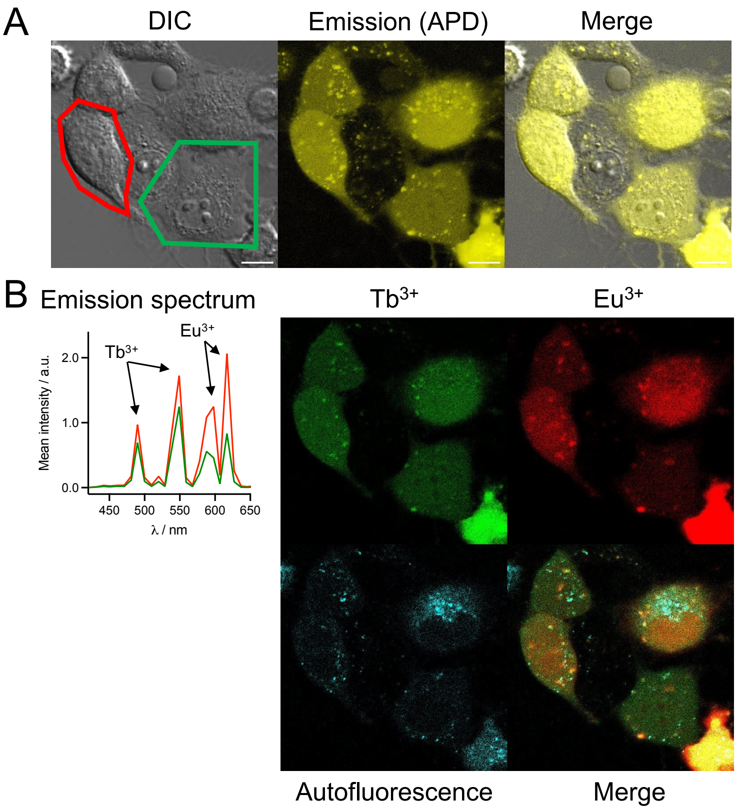

Fig. S18 2PM imaging (λ_{ex} = 720 nm) of living HeLa cells incubated 1 h with dTAT[Tb·L-^{Me}Ar-NHSuc(–)] (10 µM) and dTAT[Eu·L-Ar-NHAc] (5 µM) in PBS. (A) *Left panel:* DIC image; *Middle panel*: luminescence image recorded using APD; *Right panel:* merge. Scale bars correspond to 10 µm. (B) *Left panel:* 2P-excited emission spectra (detection with PMT) of cells outlined in red and green in panel A; *Right panel:* Tb^{3+}, Eu^{3+}, autofluorescence emission maps obtained by linear unmixing of 2P-excited spectral images recorded with the PMT and merge of the three.

Cytotoxicity

MTT proliferation assay: Inhibition of cell proliferation by dTAT[Ln·L] conjugates was measured by a MTT assay. HeLa cells were seeded into 96-well plates (3×10^3 cells per well) in 100 µL of culture medium (RPMI with 10% of fetal calf serum). After 24 h, cells were washed with PBS three times, then treated with compound dissolved at various concentrations in RPMI medium (without phenol red and without serum) during 2 h. A control with culture medium only was prepared also. Following incubation of the cells with the samples, the solution was discarded, the cells were washed twice with PBS and fresh culture medium was added to the wells. After 24 h at 37 °C, plates were centrifugated for 5 min at 400 g. The medium was then discarded and replaced with fresh culture medium containing MTT (0.5 mg/mL, Euromedex, Mundolsheim, France). After 3 h at 37 °C, 100 µL of solubilizing solution (10% Triton-X100, 0.1 M HCl, in isopropanol) were added in each well. Plates were incubated at room temperature under shaking until solubilization of water insoluble purple formazan crystals. Absorbance was then measured on an ELISA reader (Tecan, Männedorf, Switzerland) at a test wavelength of 570 nm and a reference wavelength of 650 nm. Absorbance obtained by cells in control medium was rated as 100 % cell survival. Each data point is the average of three independent experiments. Data were then fitted using GraphPad Prism to determine IC50 values.

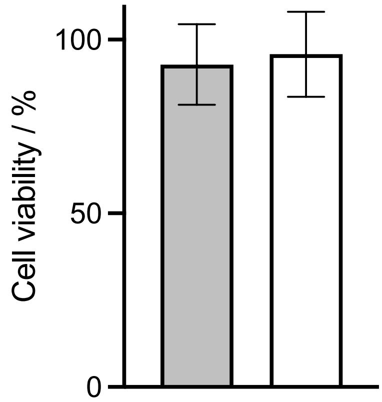

Fig. S19 Cell viability by MTT proliferation assay for co-incubations. (grey) dTAT(Tb·L-^{Me}Ar-NHβAla(+)) 10 μM + dTAT(Eu·L-Ar-NHAc) 3 μM; (white) dTAT(Tb·L-^{Me}Ar-NHSuc(–)) 10 μM + dTAT(Eu·L-Ar-NHAc) 5 μM. Error bars correspond to SD.

References

[1] B. Jagadish, T. J. Ozumerzifon, S. A. Roberts, G. B. Hall, E. A. Mash, N. Raghunand, *Synth. Commun.* **2014**, *44*, 441–449.

[2] V. Placide, A. T. Bui, A. Grichine, A. Duperray, D. Pitrat, C. Andraud, O. Maury, *Dalton Trans.* **2015**, *44*, 4918–4924.

[3] J. W. Clary, T. J. Rettenmaier, R. Snelling, W. Bryks, J. Banwell, W. T. Wipke, B. Singaram, *J. Org. Chem.* **2011**, *76*, 9602–9610.

[4] G. T. Lee, X. Jiang, K. Prasad, O. Repič, T. J. Blacklock, *Adv. Synth. Catal.* **2005**, *347*, 1921–1924.

[5] F. Mo, Y. Jiang, D. Qiu, Y. Zhang, J. Wang, *Angew. Chem., Int. Ed.* **2010**, *49*, 1846–1849.

[6] S. A. Palasek, Z. J. Cox, J. M. Collins, *J. Pept. Sci.* **2007**, *13*, 143–148.

[7] N. Thieriet, J. Alsina, E. Giralt, F. Guibe, F. Albericio, *Tetrahedron Lett.* **1997**, *38*, 7275–7278.

[8] J.-H. Choi, G. Fremy, T. Charnay, N. Fayad, J. Pécaut, S. Erbek, N. Hildebrandt, V. Martel-Frachet, A. Grichine, O. Sénèque, *Inorg. Chem.* **2022**, *61*, 20674–20689.

[9] A. Beeby, I. M. Clarkson, R. S. Dickins, S. Faulkner, D. Parker, L. Royle, A. S. de Sousa, J. A. G. Williams, M. Woods, *J. Chem. Soc., Perkin Trans. 2* **1999**, 493–504.

[10] A. M. Brouwer, *Pure Appl. Chem.* **2011**, *83*, 2213–2228.

[11] U. Resch-Genger, K. Rurack, *Pure Appl. Chem.* **2013**, *85*, 2005–2013.

[12] S. de Reguardati, J. Pahapill, A. Mikhailov, Y. Stepanenko, A. Rebane, *Opt. Express* **2016**, *24*, 9053–9066.

[13] A. Grichine, A. Haefele, S. Pascal, A. Duperray, R. Michel, C. Andraud, O. Maury, *Chem. Sci.* **2014**, *5*, 3475–3485.
